# The fern-feeder aphids (Hemiptera: Aphididae) from China: a generic account, descriptions of one new genus, one new species, one new subspecies, and keys

**DOI:** 10.1093/jis/14.1.23

**Published:** 2014-01-01

**Authors:** Xiao-Mei Su, Li-Yun Jiang, Ge-Xia Qiao

**Affiliations:** 1 Key Laboratory of Zoological Systematics and Evolution, Institute of Zoology Chinese Academy of Sciences, Beijing 100101, China; 2 Fangshan District’s Center for Animal Epidemic Disease Prevention and Control, Beijing 102400,China

## Abstract

Fern-feeder aphids (Hemiptera: Aphididae) in China are represented by 13 species in 10 genera, including a new genus, *Vietaphis***gen. nov.** , a new species, *Vietaphis aliquantus***sp. nov.** , from Guizhou and Tibet on *Plagiogyria japonicum* , and a new subspecies, *Amphorophora scabripes galba***ssp. nov.** , from Guizhou on *Pentarhizidium intermedium* . Two genera, *Amphorophora* Buckton and *Idiopterus* Davis, and four species or subspecies, *Amphorophora ampullate ben-galensis* Hille Ris Lambers and Basu, *Idiopterus nephrelepidis* Davis, *Micromyzodium polypodii* Takahashi, and *Myzus filicis* Basu, are reported for the first time in China. Apterae and alatae of *Myzus filicis* are redescribed herein, and with host plant notes. The fern-feeder aphid genus *Ne-omacromyzus* Lee is considered a junior synonym of *Idiopterus* . Furthermore, *Neomacromyzus cyrtomicola* Lee is transferred to the genus *Idiopterus* , as *Idiopterus cyrtomicola* (Lee), **comb. nov.** , which is herein considered a junior synonym of *Idiopterus nephrelepidis* Davis. Keys to Chinese fern-feeder species are provided. Morphological figures and biometrical data of *Vietaphis aliquantus***sp. nov.** , *Amphorophora scabripes galba***ssp. nov.** , and *Myzus filicis* are presented.

## Introduction

Aphids feeding on ferns are few compared to ones on other host plants, such as gymno-sperms, herbaceous monocotyledons, or various angiosperm families. Robinson (1966) reviewed 11 species of fern-feeding aphids (Hemiptera: Aphididae) in North America. Subsequently, Miyazaki (1968) reported 10 fern-feeding aphid species distributed in Japan. Jensen and Holman (2000) provided an accurate list of fern-feeding aphids, including 44 species belonging to 14 genera in the world. Thereafter, studies on fern aphids have been limited and only confined to descriptions of a few new species or new genera ( Lee 2006 ; Su and Qiao 2010 ).

Among aphid samples collected from South and Southwest China, as part of an ongoing survey of Aphididae in Guizhou and Tibet, fern-feeding aphid samples were obtained. Based on these samples, the fern-feeding aphids from China are herein reviewed. Currently, the fern-feeding aphid fauna of China is represented by 13 species or subspecies belonging to 10 genera.

The original description of the apterae of *Myzus filicis* Basu was very simple ( Basu 1969 ). Here, based on material collected in China, the apterous viviparous female and the hitherto unknown alatae of *M. filicis* are described in detail. While checking specimens and the literature, we found that *Neomacromyzus* Lee is a junior synonym of the genus *Idiopterus* Davis. As a result, *Neomacromyzus cyrtomicola* Lee is transferred to *Idiopterus* , as *Idiopterus cyrtomicola* (Lee), **comb. nov.** , which is herein considered a junior synonym of *Idiopterus nephrelepidis* Davis.

## Materials and Methods

The host plants of all samples of the new species were determined by the staff of Prof. L. Q. Li and Prof. X. C. Zhang, Institute of Botany, Chinese Academy of Sciences, Beijing, China.

Aphid terminology in this paper generally follows Heie (1994) and Qiao et al. (2006) . The measurements are in millimeters.

Specimen depositories: the specimens examined in this study, including types, are deposited in the National Zoological Museum of China, Institute of Zoology, Chinese Academy of Sciences, Beijing, China, except the specimens of *Micromyzus judenkoi* Carver in the Natural History Museum, London, U.K. (BMNH).

Abbreviations in Tables: **Ant. I, II, III, IV, V, VIb** : antennal segments I, II, III, IV, V, and the base of Ant. VI, respectively; **PT** : processus terminalis; **b.d.III** : basal diameter of antennal segment III; **URS** : ultimate rostral segment; **BW URS** : basal width of ultimate rostral segment; **MW hind tibia** : middle diameter of hind tibia; **BW SIPH** : basal width of siphunculus; **SIPH** : siphunculus; **DW SIPH** : distal width of siphunculus; **BW Cauda** : basal width of cauda; **Setae on Tergite I** : marginal setae on abdominal tergite I; **Setae on Tergite VIII** : spinal setae on abdominal tergite VIII.

### Nomenclature

This publication and the nomenclature it contains have been registered in ZooBank. The LSID number is:

urn:lsid:zoobank.org:pub:BFF56AE4-4EF7-43FE-84B3-C1EAB114C0AA. It can be found online by inserting the LSID number after www.zoobank.com/ .

### Taxonomic account

**Key to genera of fern-feeding aphids in China**

**Figure 1.**
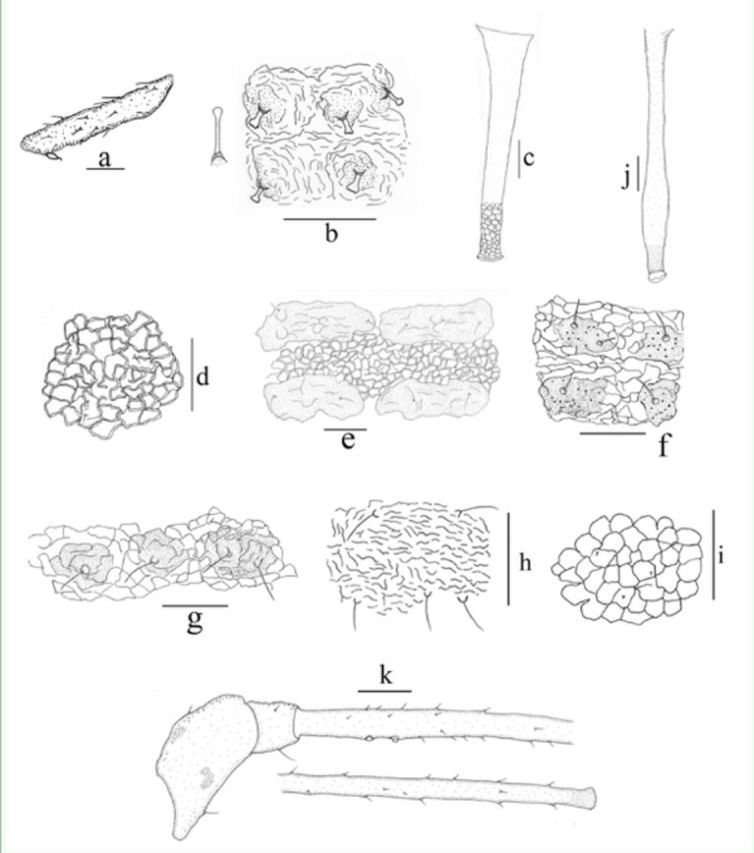
Apterous viviparous female: **a.** hind tibia and tarsus of *Shinjipteridifoliae* . **b.** dorsal capitate or flabellate setae of body of *Idiopterusnephrelepidis* . **c** . siphunculus of *Macromyzus woodwardiae* . **d.** body dorsalrecticulations of *Macromyzella polypodicola* . **e.** pairs of spinalsclerites onabdominal tergites of *Macromyzus maculatus* . **f.** body dorsum with hairbearingsclerites of *Macromyzus spinosus* (big spinules shown). **g** . bodydorsum with hairbearing sclerites of *Macromyzuswoodwardiae* . **h** . thickand long dorsal setae of body of *Micromyzodium polypodii* . **i.** C- or Oshapedwrinkles on abdominal tergites of *Myzus filicis* . **j.** siphunculus of *Amphorophora ampullate bengalensis* . **k** . antennal segment III of *Amphorophoraampullata bengalensis* . Scale bars = 0.10 mm.

1. Tarsi atrophied and without claws; antennae 6-segmented; host-alternating between *Viburnum* and ferns ( Figure1a )……………. *Shinjia* Tarsi normal, with 2 claws………………2

2.Dorsal setae of body capitate or flabellate ( Figure 1b ); antennal tubercles developed, parallel or slightly diverging at inner sides; 1/3 basal part of siphunculus brown, others pale………...…………... *Idiopterus* Dorsal setae of body normal, acute or blunt, but not capitate nor flabellate…….3

3.Distal part of siphunculus with reticulations ( Figure 1c ); dorsum of body with distinct reticulations ( Figures 1d-g )…….4 Distal part of siphunculus without reticulations; dorsum of body without distinct reticulations……………………………...5

4. Antennal segment III always without secondary rhinaria; dorsal setae of body short with acute to incrassate apices, always less than basal diameter of antennal segment III, without sclerites at bases ( Figure 1d )………………………… *Macromyzella* Antennal segment III with or without secondary rhinaria; dorsal setae of body long and blunt, usually more than basal diameter of antennal segment III, with tubercles or sclerites at base ( Figures 1e-g )……….. ……...……………………… *Macromyzus*

5.Dorsal setae of body thick and long, usually as long as or more than basal diameter of antennal segment III ( Figure 1h ); processus terminalis 5.50-7.60 times as long as base of segment VI….. *Micromyzodium* Dorsal setae of body short, usually less than basal diameter of antennal segment III………………………………………Antennal tubercles highly developed, and distinctly converging at inner sides………………………………. *Myzus* Antennal tubercles distinct, but parallel or diverging at inner sides………………….7

**Figure 3.**
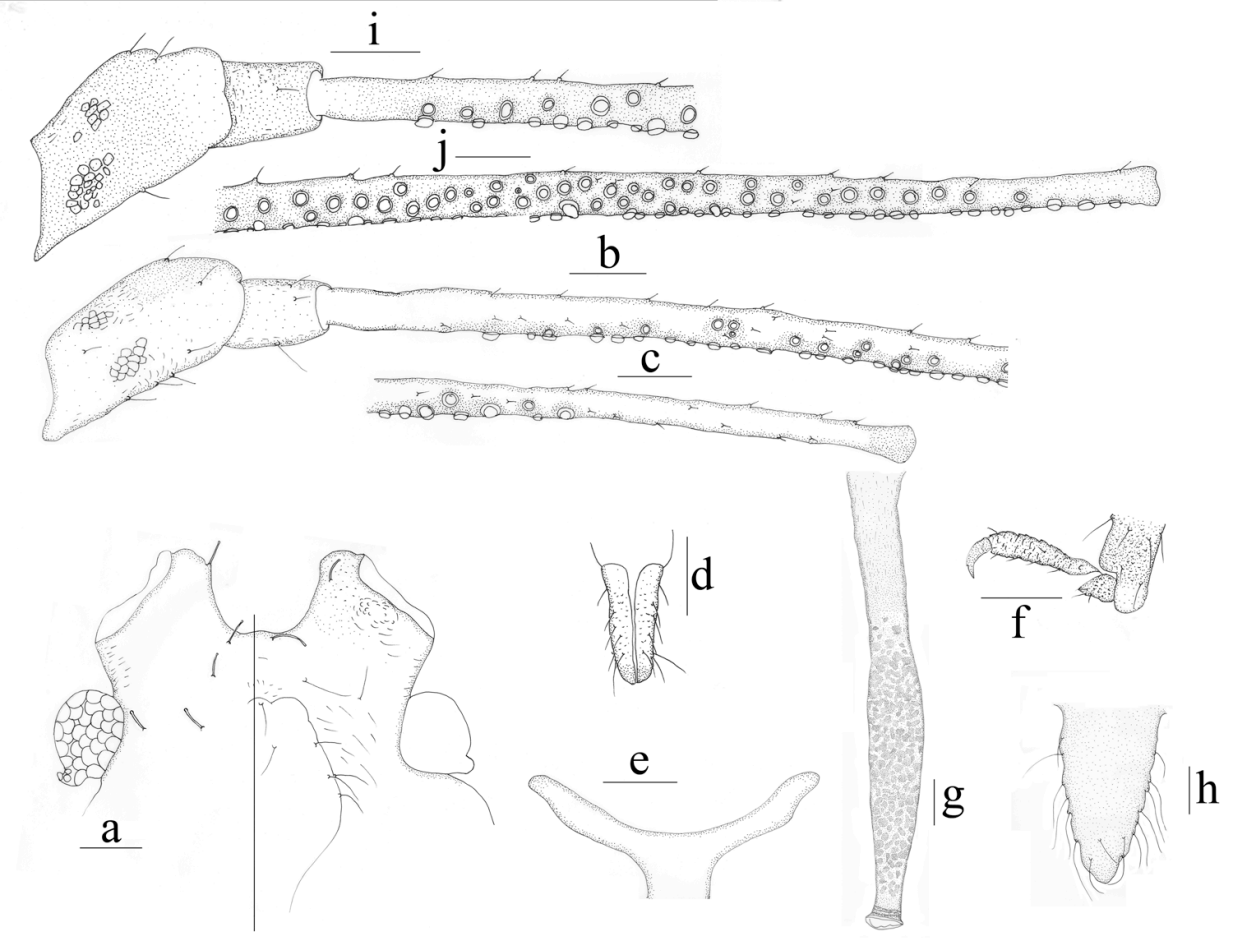
*Amphorophora scabripes galba* ssp. nov., (a–h) apterous viviparousfemale: **a** . dorsal (left) and ventral (right) view of head. **b–c.** antennal segments I–III. **d** . ultimate rostral segment. **e** . mesosternalfurca. **f.** hind tarsus (spinules on first tarsal segment shown). **g** . siphunculus(different degree pigmented patches shown). **h** . cauda. **i–j.** alateviviparous female: **i.** antennal segments I–II and basal part of segment III. **j.** rest part of antennal segment III. Scale bar: 0.10 mm.

**Figure 4.**
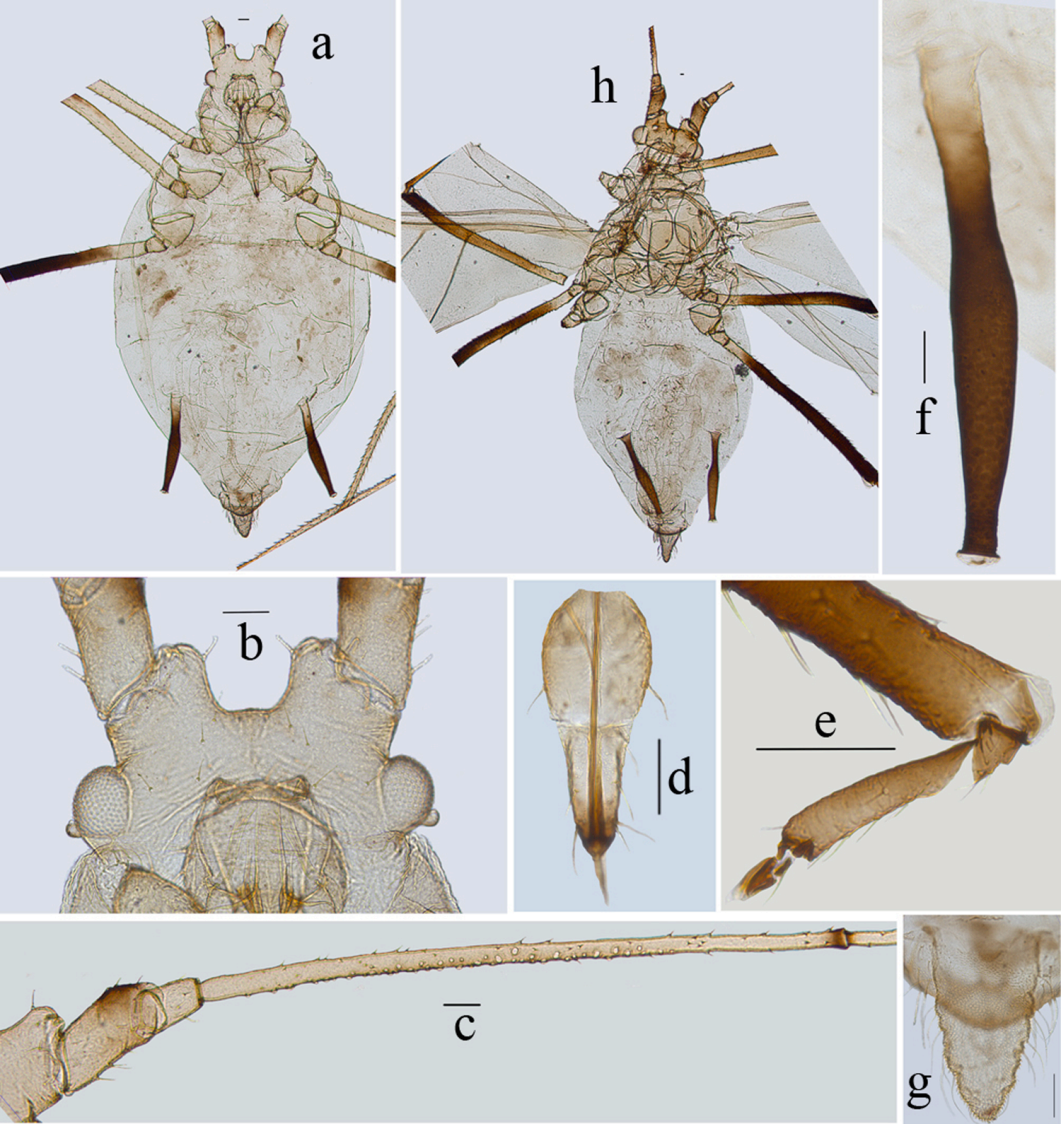
*Amphorophora scabripes galba* **ssp. nov** . ( **a–g** ) apterousviviparous female: **a.** dorsal view of body. **b.** dorsal view of head. **c** antennal segments I–III. **d.** ultimate rostral segment. **e.** end of the hindtibia and tarsus (spinules on first tarsal segment shown). **f.** siphunculus. **g** . cauda. **h.** dorsal view of body of alate viviparous female. Scale bars =0.10 mm.

6.Siphunculi distinctly swollen, the swelling width 1.30-1.70 times as long as width of the narrowest part ( Figures 1j , 3g , 4f ). Dorsum of head smooth; distal part of femora or tibiae usually with imbrications or spinules; with shorter setae, which are without sclerites at base….. *Amphorophora* Siphunculi cylindrical or slightly swollen, but if swelling, then the swelling width at most 1.10-1.20 times as long as width of the narrowest part……………………….8

7.Dorsum of head smooth, except near eyes with C-shaped wrinkles; dorsum of body with C- or O-shaped wrinkles; wing viens normal, without border…………...……… …………………. *Vietaphis***gen.nov.** Dorsum of head spinulose, at least on marginal areas; dorsum of body smooth, or weakly reticulate, if with wrinkles, but no C- and O-shaped; wing veins dark brown or strongly bordered……………..9

8.First tarsal segments usually with 4 setae; abdominal tergites without marginal tubercles; alatae without a dark dorsal central patch on abdomen…… *Micromyzus* First tarsal segments usually with 3 setae; abdominal tergites I-VI usually with marginal tubercles; alatae with a dark dorsal central patch on abdomen… *Micromyzella*

***Amphorophora* Buckton** (new record for China)

*Amphorophora* Buckton, 1876 : 187. Type species: *Amphorophora ampullata*Buckton, 1876 .

*Eunectarosiphon* del Guercio, 1913: 188. Type species: *Aphis rubi*Kaltenbach, 1843 . *Rhopalosiphum*van der Goot, 1913 : 146, nec Koch, 1854. Type species: *Amphorophora ampullata*Buckton, 1876 .

***Comment*** : Two species of the genus, i.e., *A. ampullata* Buckton and *A. scabripes* Miyazaki, are fern-feeders. *Amphorophora ampullata* is being applied to a complex of species with different fern associations.

On *A. ampullata* , the populations in America are regarded as a subspecies, *A* . *ampullata laingi* Mason, and mainly associated with *Onoclea sensibilis* and *Matteuccia* spp. (Onocleaceae), and the ones in Northeast India and Nepal are regarded as another subspecies, *A. ampullata bengalensis* Hille Ris Lambers & Basu, which colonize various ferns (Blackman and Eastop 2006). Here, *A. ampullata bengalensis* is recognized for the first time in China.

Miyazaki (1968) described another fern-feeding aphid species of the genus *Amphorophora* , namely *A. scabripes* . Comparing with the original description of the species, some specimens from Guizhou, China, on *Pentarhizidium intermedium* are very close to this species, but differ from it in the number of secondary rhinaria on antennal segment III, the ratio of ultimate rostral segment to second segment of hind tarsus. So, these specimens are herein regarded as a new subspecies, *A. scabripes galba***ssp. nov.** The new subspecies lives on the undersides of fronds of ferns. Thus, there are two subspecies of fern-feeders in the genus from China.

**Key to fern-feeding aphid species of** *Amphorophora* **in China based on apterous viviparous females**

1. Antennal segment III with 2–9 secondary rhinaria ( Figure 1k ); ultimate rostral segment 1.27–1.43 times as long as second hind tarsal segment………………………. …... *Amphorophora ampullata bengalensis* - Antennal segment III with 21–50 secondary rhinaria ( Figures 3b , 4c ); ultimate rostral segment 0.96–1.12 times as long as second hind tarsal segment………………. ... *Amphorophora scabripes galba***ssp. nov.**

**Figure 2.**
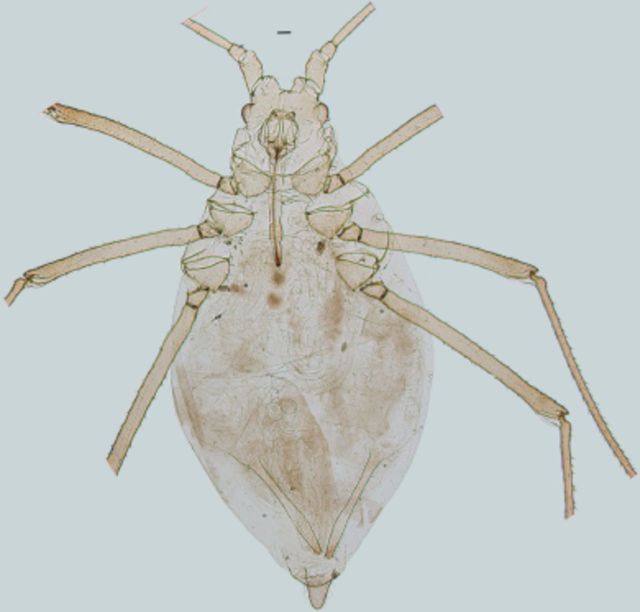
Apterous viviparous female of *Amphorophora ampullata bengalensis* .Scale bar = 0.10 mm

*Amphorophora ampullata bengalensis* **Hille Ris Lambers & Basu** (new record for China) ( Figure 2 )

*Amphorophora ampullata bengalensis* Hille Ris Lambers & Basu, 1966 : 14.

*Amphorophora ampullata bengalensis* Hille Ris Lambers & Basu: Eastop & Hille Ris Lambers, 1976 : 23; Raychaudhuri, 1980 : 96; Ghosh, 1986: 54; Remaudière & Remaudière, 1997 : 71; Blackman & Eastop, 2006: 1039.

***Material examined*** : **China** . **Guangdong** : 4 apterous viviparous females, Guangzhou City (E113.13°, N238.17°), altitude 7 m, 26.iii.1984, on a kind of fern, by B.Y. Ye (No. Y7857); 1 apterous viviparous female, Ruyuan County (E113.08°, N25.10°), altitude 1399 m, 11.vii.2008, on a kind of fern, by X.M. Su (No. 21790); **Fujian** : 2 apterous viviparous females, Wuyishan Mountain (E109.48°, N30.27°), altitude 461 m, 4.vii.2003, on a kind of fern, by X.L. Huang (No. 14403); 1 apterous oviparous female and 3 apterous viviparous females, Meihuashan Mountain (E116.09°, N25.29°), altitude 1130 m, 4.xi.2008, on a kind of fern, by H.H.

Zhang and F.Q. Chen (No. 22025); **Hunan** : 2 apterous viviparous females, Liling County (E116.09°, N25.29°), altitude 868 m, 7.vii.2008, on a kind of fern, by X.M. Su (No. 21730); 2 apterous viviparous females, Guidong County (E113.07°, N25.10°), altitude 1143 m, 10.vii.2008, on a kind of fern, by X.M. Su (No. 21758); 1 apterous viviparous female, Yizhang County (E112.10°, N24.10°), altitude 1572 m, 14.vii.2008, on a kind of fern, by X.M. Su (No. 21814); **Tibet** : 1 alate nymph, 1 apterous nymph, Yadong County (E88.9°, N27.48°), altitude 3747 m, 17.viii.2010, on *Dryopteris chrysocoma* , by Y. Wang (No. 25925).

**Figure 17.**
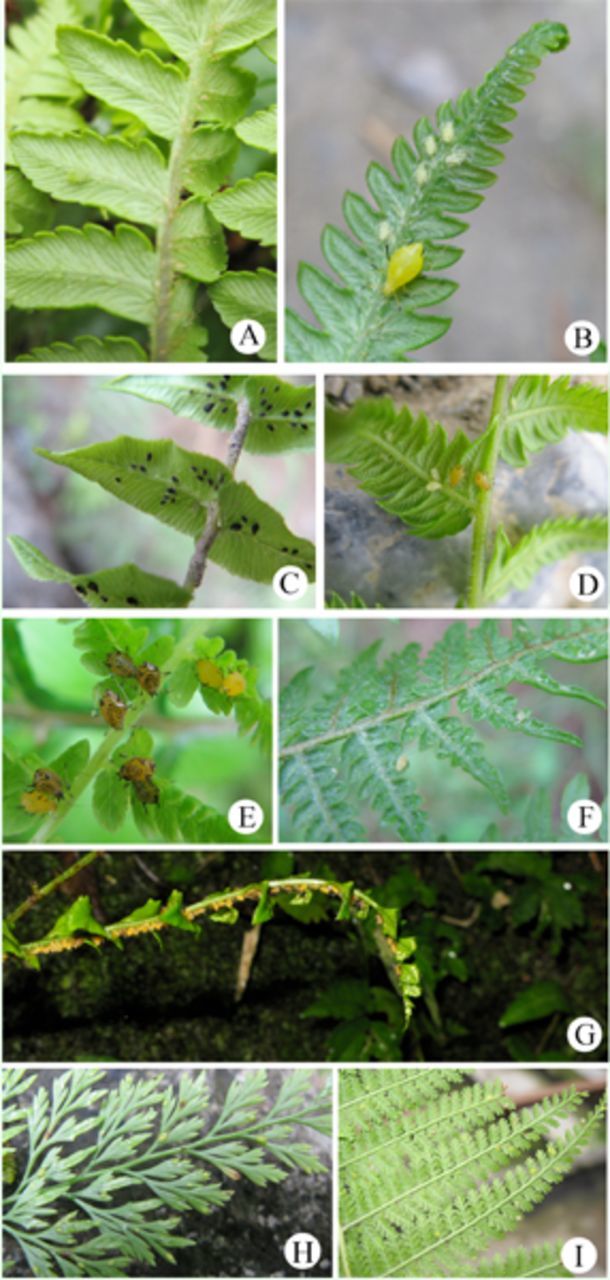
The ecological photos in the field. **A** . alate nymph of *Amphorophoraampullate bengalensis* . **B** . colonies of *Amphorophora scabripesgalba***ssp. nov***.***C** . colonies of *Idiopterus nephrelepidis* . **D** .colonies of *Macromyzella polypodicola* . **E** . colonies of *Macromyzus maculatus* . **F** . smallcolonies of *Shinjia orientalis* . **G** .coloniesof *Macromyzuswoodwardiae* . **H** .colonies of *Vietaphis aliquantus***sp. nov. I** . colonies of *Myzus filicis* .

***Host plants*** : *Athyrium* sp *.* (Athyriaceae) (Raychaudhuri 1974); *Asplenium* sp *.* (Aspleniaceae), *Cheilanthes* sp. (Sinopteridaceae) (Ghosh 1974; Raychaudhuri 1974); *Pteridium aquilinum* (Pteridiaceae) ( Ghosh, Ghosh and Raychaudhuri 1970 ); *Polypodium* sp. (Polypodiaceae) ( Raychaudhuri 1980 ). *Dryopteris chrysocoma* (Dryopteridaceae) is newly added to the list of host plants ( Figure 17A ).

***Distribution*** : China (Guangdong, Fujian, Hunan, Tibet), India, and Nepal.

***Biology*** : This aphid colonizes on ferns ( Figure 17A ). The oviparous female still can be observed in November in South China.

*Amphorophora scabripes galba* **ssp. nov** *.* ( Table 1 and Figures 3–4)

**Table 1.**
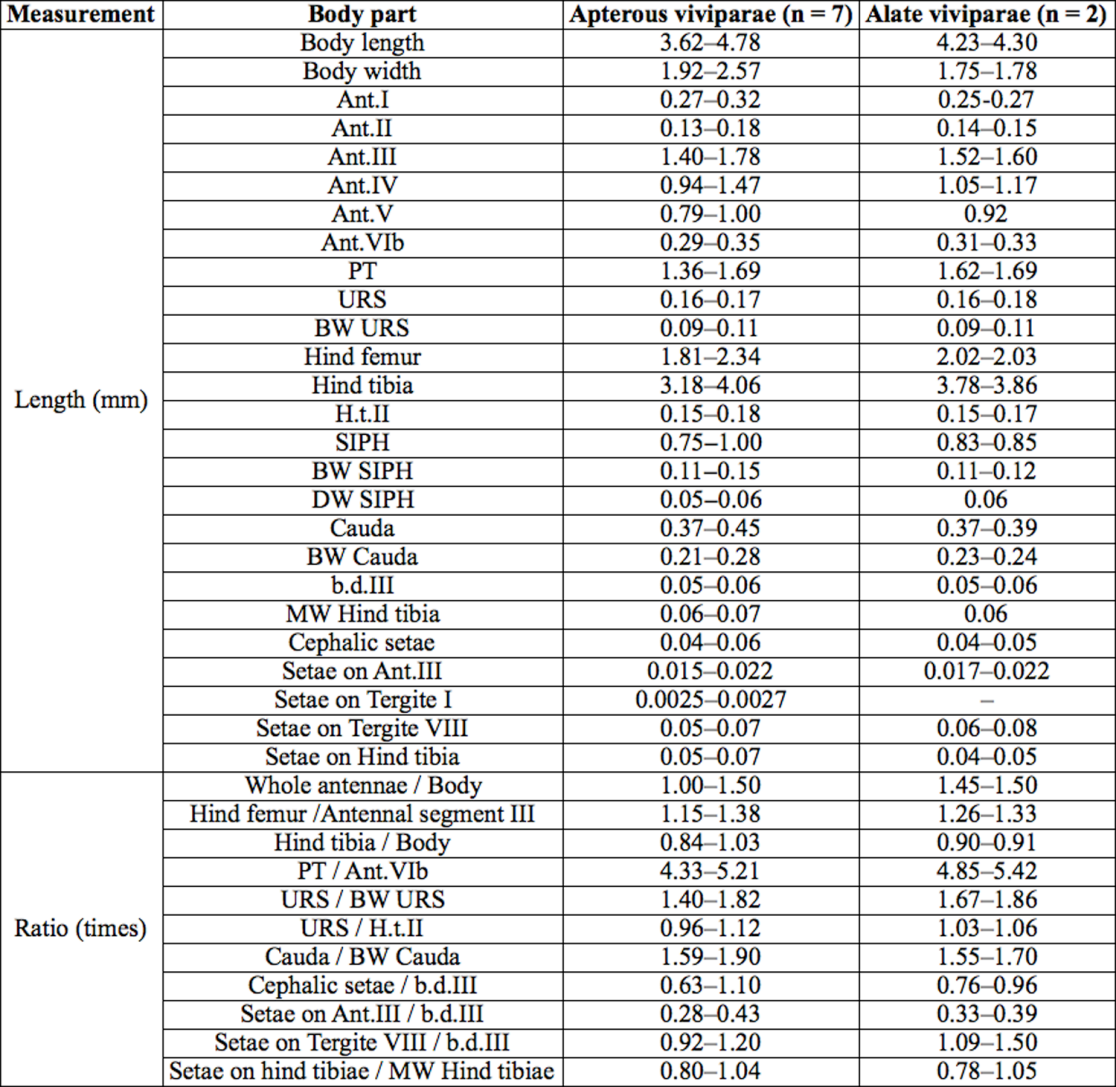
Biometric data (mean, range) of *Amphorophora scabripes galba***ssp. nov.** (in mm).

***Type locality*** : China (Guizhou, E 107.1°, N28.2°, altitude 1470 m).

***Etymology*** : The species name consists in “ *galbus* ” (= yellow in Latin). The species is named after its yellow body color when it is alive.

***Description*** : *Apterous viviparous female:* Body elongated oval, yellow, antennae, legs and siphunculi dark brown when alive ( Figure 17B ).

***Mounted specimens*** : Body pale brown ( Figure 4a ), 3.62–4.78 mm long and 1.92–2.57 mm wide. For general measurements see Table 1 .

*Head* : slightly brown, smooth or faintly wrinkled dorsally, and sparse spinules near antennal tubercles ventrally ( Figures 3a , 4b ). Median frontal tubercle slightly prominent; antennal tubercles developed, slightly diverging at inner sides; with 2 pairs of antennal tubercles setae and a pair of median frontal setae. Dorsal setae of head long and incrassate apices, with 2 pairs of setae between antennae, arranged longitudinally, and 2 pairs of setae between compound eyes, arranged transversely. Cephalic setae 0.80–1.00 times as long as basal diameter of antennal segment III. Ventral setae similar to dorsal ones with acute apices. Eyes large with distinct ocular tubercles. Antennae 6-segmented, inner margin of antennal segment I, apex of antennal segment III, secondary rhinaria around, apices of antennal segments IV–V, and the base of segment VI distally dark brown, antennal segments I and II spinulose ventrally and smooth dorsally, segment III smooth; segments IV–VI imbricated, ones on segment IV weak ( Figures 3b , c , 4c ). Antennae 1.00–1.50 times as long as body, length in proportion of segments I–VI: 17–19, 9–10, 100, 67–82, 56– 57, 18–23+94–103, processus terminalis 4.33– 5.21 times as long as the base of the segment. Antennal setae thick, and short, segments I– VI each with 12–14, 5 or 6，38–40，22–24 ，11–14，10–12+3 setae, respectively, apex of processus terminalis with 3 setae; setae on segment III 0.28–0.43 times as long as basal diameter of the segment. Primary rhinaria ciliated, segment III with 21-50 round and proberant secondary rhinaria. Rostrum reaching beyond middle coxae, ultimate rostral segment wedge-shaped, apex dark brown and spinulose ( Figures 3d , 4d ), 1.40-1.82 times as long as its basal width, 0.96-1.12 times as long as second segment of hind tarsus, with 6 primary setae and 8 secondary setae.

*Thorax:* legs long. Fore and middle femora distally, and hind femora dark brown except for 1/5 basal part pale, distal parts of tibiae and tarsi dark brown. Coxae with spinules ventrally, femora and tibiae distally spinulose, first tarsal segments spinulose ( Figures 3f , 4e ), others smooth. Hind femora 1.15-1.38 times as long as antennal segment III. Hind tibiae 0.84-1.03 times as long as body, setae on hind tibiae thick, long and incrassate apices, 0.80-1.04 times as long as middle diameter of the segment. First tarsal chaetotaxy: 3, 3, 3. Hind tibiae of nymph with spinules.

*Abdomen:* abdominal tergites I-VI smooth, posterior areas of siphunculi, tergites VII and VIII with spinules. Venter with transverse rows of spinules. Spiracles nephroid, spiracular plates pale brown and slightly prominent. Dorsal setae on abdominal tergites I-VI thick and short, with blunt apices, setae on tergites VII and VIII a little longer than anterior ones. Ventral setae long and acute. Tergites VIII with 8 long setae. Siphunculi dark brown except for 1/6-1/7 basal part pale brown, and with different degree pigmented patches, cylindrical, slightly unsymmetrical swollen on distal half, sparse wrinkled, with two rows of corrugates under developed flange ( Figures 3g , 4f ), 0.19-0.21 times as long as body, 5.67-6.81 times as long as its basal width, the widest part 0.77-1.08 times as long as its basal width, 2.03-2.22 times as long as cauda. Cauda pale, thick coniform, distal part slightly blunt ( Figures 3h , 4g ), with 20-24 setae. Anal plate pale, transverse oval, with 10 setae. Genital plate pale, broad round with 12-14 posterior setae and 4 anterior setae.

***Alate viviparous female** :* Body yellow with dark brown head, thorax, legs and siphunculi in life.

*Mounted specimens:* body pale brown ( Figure 4h ), 4.23-4.30 mm long and 1.75-1.78 mm wide. For general measurements see Table 1 .

*Head:* slightly brown and smooth with sparse wrinkles dorsally and ventrally. Antennal tubercles developed, slightly diverging at inner sides. Dorsal setae of head long and blunt, 0.76-0.96 times as long as basal diameter of antennal segment III. Antennae 6-segmented, brown except for basal part of segment III pale. Antennae 1.45-1.50 times as long as body, length in proportion of segments I-VI: 16-17 **,** 9 **,** 100 **,** 73-76 **,** 58-61 **,** 20-22+105-107, processus terminalis 4.85-5.42 times as long as the base of the segment. Primary rhinaria ciliated, segment III with 85-90 large secondary rhinaria ( Figs 3i , 3j ). Setae on segment III 0.33-0.39 times as long as basal diameter of the segment. Apex of rostrum dark brown and spinulose, 1.67-1.86 times as long as its basal width, 1.03-1.06 times as long as second segment of hind tarsus, with 6 primary setae and 8 accessory setae.

*Thorax:* legs long, femora brown except for basal part pale, distal parts of tibiae, tarsi dark brown. Coxae with spinules ventrally, 1/5 distal parts of femora, distal parts of tibiae and first tarsal segments spinulose, others smooth. Hind femora 1.26-1.33 times as long as antennal segment III. Hind tibiae 0.90-0.91 times as long as body; setae on hind tibiae 0.78-1.05 times as long as middle diameter of the segment. First tarsal chaetotaxy: 3, 3, 3.

*Abdomen:* abdominal tergites smooth. Abdominal tergites II-IV with very pale marginal sclerites. Dorsal setae similar to ones of apterae. Tergite VIII with 8 long setae. Siphunculi 0.19-0.20 times as long as body, 6.96-7.39 times as long as its basal width, the widest part 0.90-0.93 times as long as its basal width, 2.18-2.24 times as long as cauda. Cauda pale brown, slightly thinner than ones of apterae, with 20-24 setae. Anal plate with 10-14 setae. Genital plate with 12 posterior setae and 4 anterior setae. Wing veins normal. Others are similar to apterae.

***Holotype** :* apterous viviparous female, **China** . **Guizhou** : Zunyi City (E107.1°, N28.2°), altitude 1470 m, 6.vi.2010, on *Pentarhizidium intermedium,* by X.M. Su (No. 24557).

***aratypes** :* **China** . **Guizhou** : 1 alate viviparous female, same data as holotype (No. 24557); 1 apterous nymph, 1 alate viviparous female, 1 apterous viviparous female, Zunyi City (E107.1°, N28.2°), altitude 1470 m, 7.vi.2010, *on Pentarhizidium intermedium,* by X.M. Su (No. 24562, 24564); 2 apteous viviparous females, Zunyi City (E107.1°, N28.2°), altitude 1470 m, 14.viii.2010, *on Pentarhizidium intermedium,* by X.M. Su (No. 25627); 2 apteous viviparous females, Zunyi City (E107.1°, N28.2°), altitude 1470 m, 15.viii.2010, on *Pentarhizidium intermedium,* by X.M. Su (No. 25628).

***Host plant** : Pentarhizidium intermedium* (Onocleaceae).

***Distribution** :* China (Guizhou).

***Biology** :* The species infests scatteringly on the undersides of the distal parts of fronds of fern, and without causing distinct injury ( Figure 17B ). The species was not attended by ants.

***Comment*** : The new subspecies is very closed to the nominate subspecies, *Amphorophora scabripes scabripes* Miyazaki, but differs from the latter in: (1) apterae yellow with dark brown antennae, legs and siphunculi except for basal part pale in life (the latter: apterae pale green with a pair of longitudinal dark green stripes dorsally, and black-tipped siphunculi), (2) antennal segment III with 21– 50 secondary rhinaria in apterae, 85–90 ones in alatae (the latter: antennal segment III with 19–27 ones in apterae, 53–69 ones in alatae), (3) ultimate rostral segment with 8 accessory setae (the latter: with 4–6 accessory setae), (4) cauda with 20–24 setae (the latter: with 15–20 setae)(the material of the nominate subspecies from Miyazaki 1968 ).

*Idiopterus* **Davis** (new record for China)

*Idiopterus* Davis, 1909 : 198. Type species: *Idiopterus nephrelepidis*Davis, 1909*Fullawayella*del Guercio, 1911 : 462. Type species: *Macrosiphum kirkaldyi*Fullaway, 1910 .

*Neomacromyzus* Lee, 2006 : 493. Type species: *Neomacromyzus cyrtomicola*Lee, 2006 .

***Comment*** : The genus is a monotypic genus for one fern-feeding species, *Idiopterus nephrelepidis* Davis. Lee (2006) reported a new fern-feeding aphid genus *Neomacromyzus* Lee and a new species, *Neomacromyzus cyrtomicola* Lee on *Cyrtomium falcatum* from the southernmost region of Korea, but based on the original description, the genus *Neomacromyzus* Lee is herein considered a junior synonym of *Idiopterus* Davis. As a result *Neomacromyzus cyrtomicola* Lee is transferred to the genus *Idiopterus* as *Idiopterus cyrtomicola* (Lee) **comb. nov.** , which is herein considered as a junior synonym of *Idiopterus nephrelepidis* Davis.

**Figure 5.**
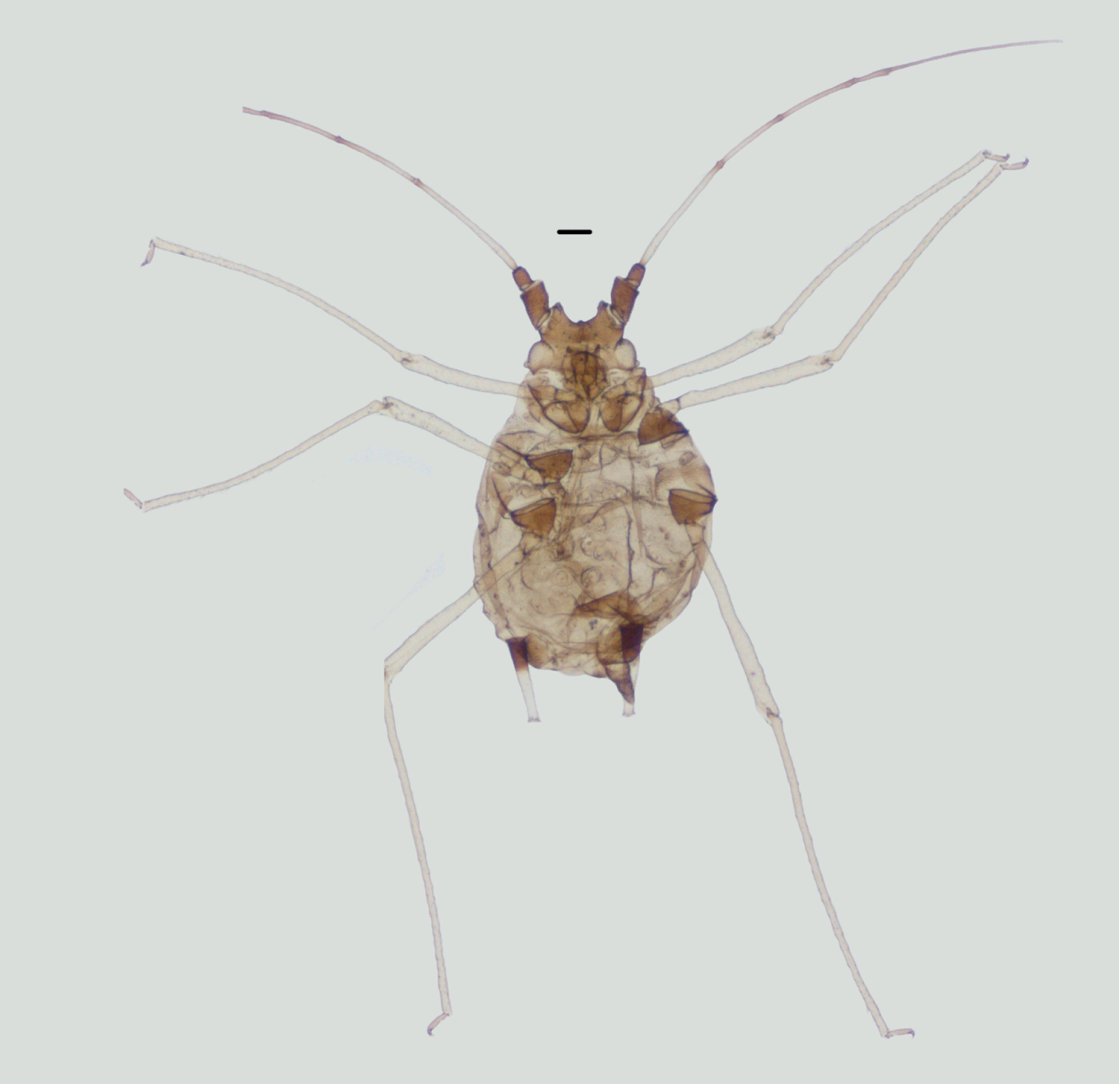
Apterous viviparous female of *Idiopterus nephrelepidis* . Scale bars = 0.10 mm.

*Idiopterus nephrelepidis* **Davis** (new record for China) ( Figure 5 )

*Idiopterus nephrelepidis* Davis, 1909 : 199. *Macrosiphum kirkaldyi*Fullaway, 1910 : 462. *Neomacromyzus cyrtomicola*Lee, 2006 : 493.

### Syn. nov.

*Idiopterus nephrelepidis* Davis: Heie, 1994 : 83; Eastop & Hille Ris Lambers, 1976 : 223; Remaudière & Remaudière, 1997 : 102; Blackman & Eastop, 2006: 1174.

***Material examined** :* **China** . **Chongqing** : 4 apterous viviparous females, Jinfoshan Mountain (E107.15°, N29.02°), altitude 1906 m, 14.vi.2010, on *Pteris vittata,* by F.Q. Chen (No. 24608).

***Host plants** : Asplenium* sp., *Pteridium* spp and *Polypodium* sp. ( Heie 1994 ); *Cyrtomiumfalcatum* (Dryopteridaceae) ( Lee 2006 ). *Adiantum edgeworthii A. fritz-luedtii, A.hispidulum, A. pedatum* (Adiantaceae); *Anthu-rium andreanum* (Araceae); *Aphelandra squarrosa* (Acanthaceae); *Aspidium trifoliatum* (Aspidiaceae); *Blechnum patersonii*

(Blechnaceae); *Crocus* sp. (Iridaceae); *Cyrtomium falcatum* (Aspidiaceae); *Dryopterisfilix-mas* (Aspidiaceae); *Davallia bullata* (Da-valliaceae), *Dennstaedtia adianthoides* (Dennstaedtiaceae), *Microlepia speluncae* (Dennstaedtiaceae), *Pellaea hastata* (Sinopteridaceae), *Phyllitisscolopendrium*

(Aspleniaceae), *Platycerium alcicorne* (Polypodiaceae), *P. aureum, P. lycopodioides, P. piloselloides* (Polypodiaceae); *Polystichum plumosum densum, P. setiferum* (Aspidiaceae); *Pteris childsii, P. cretica, P. pancheris, P. roeveni?, P. wimsetti?* (Pteridaceae); *Saintpaulia ionantha* (Gesneriaceae), *Saxifraga stolonifera* (Saxifragaceae), *Sedum dasyphyl-lum* (Crassulaceae), *Streptocarpus hybridus* (Gesneriaceae), *Tulipa* sp. (Liliaceae), *Zan-tedeschia aethiopica* (Araceae) ( Iglisch1963 ). *Blechnum occidentale* (Blechnaceae), *Davallia canariensis* , *D. platyphylla* (Daval-liaceae); *Nephrodium* sp. (Aspidiaceae), *Nephrolepis* sp. (Oleandraceae), *Polypodiumrevolutum* (Polypodiaceae), (Hille Ris Lambers 1949). *Adonis* sp. (Ranunculaceae), *Asplenium adiantum-nigrum* (Aspleniaceae), *Nephrolepis exaltata* (Oleandraceae), *Pteris* sp. (Pteridaceae) ( Börner 1952 ). *Adiantumcapillus-veneris* (Adiantaceae) ( Leclant and Remaudière 1974 ). *Asplenium* sp. (Aspleniaceae) ( Hodjat 1998 ); *Asplenium adiantum-nigrum* (Aspleniaceae) ( Börner 1952 ; Eastop1953 ). *Asplenium hemionitis* (Aspleniaceae), *Polypodium* sp. (Polypodiaceae) ( Nieto Nafríaet al. 1984 ). *Asplenium marinum* (Aspleniaceae) ( Shaw 1964 ). *Asplenium nidus* , *A. ruta-muraria* (Aspleniaceae) *Pteris tremula* (Pteridaceae), *Onychium japonicum* (Orchidaceae)( Werder 1931 ); *Ceterach officinarium* (Aspleniaceae), *Asplen trichomanes* (Aspleniaceae), *Dryopteris* sp. (Aspidiaceae)( Steffan 1962 ). *Pteris vittata* (Pteridaceae) isnewly added to the list of the host plants ( Figure 17C ).

***Distribution*** : China (Chongqing); perhaps Neotropical in origin ( Holman 1974 ), now almost cosmopolitan, but confined to glasshouses and caves in northern temperate regions (Blackman and Eastop 2006).

***Biology*** : The species feeds on the undersides of fronds of ferns ( Figure 17C ), and anholocyclic ( Heie 1994 ). The species was not seen attended by ants.

*Macromyzella* **Ghosh, Basu & Raychaudhuri**

*Macromyzella* Ghosh, Basu & Raychaudhuri, 1977 : 582. Type species: *Myzus polypodicola*Takahashi, 1921 .

***Comment** :* The genus is found infesting fern species and is mainly distributed in East Asia. The genus is related to *Macromyzus,* but differs from it in: members of the genus with shorter setae, which are without sclerites at base, and antennal segment III always without secondary rhinaria.

**Figure 6.**
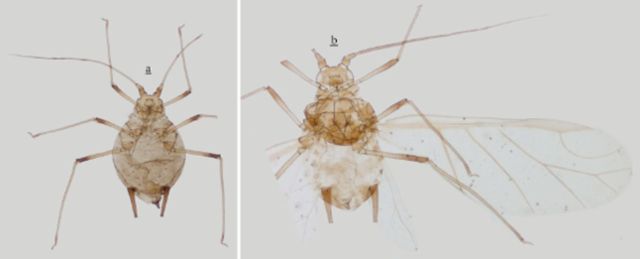
*Macromyzella polypodicola* . **a.** apterous viviparous female. **b.** alate viviparous female. Scale bars = 0.10 mm.

*Macromyzella polypodicola* **(Takahashi)** ( Figure 6 )

*Myzus polypodicola* Takahashi, 1921 : 21. *Macromyzella polypodicola* (Takahashi): Raychaudhuri, 1980 : 161; Remaudière & Remaudière, 1997 : 102; Noordam, 2004 : 99; Blackman & Eastop, 2006: 1191.

***Material examined** :* **China. Taiwan** : 3 alate viviparous females, E 120.95°, N23.7°, altitude 1090 m, 21.iii.1961, on a kind of fern, by E.I. Schhiqer (No. Y7956); **Hubei** : 5 alate viviparous females, 2 apterous viviparous females, Enshi City (E109.48°, N30.27°), altitude 461 m, 22.x.1977, on a kind of fern, by G.X. Zhang (No. 6703); **Fujian** : 1 apterous viviparous female, Wuyishan Mountain (E109.48°, N30.27°), altitude 461 m, 6.vii.2003, on a kind of fern, by X.L. Huang (No. 14427); 1 alate viviparous female, 3 apterous viviparous females, Wuping County (E116.19°, N25.2°), altitude 579 m, 19.xi.2008, on a kind of fern, by H.H. Zhang (No. 22136); **Guangxi** : 2 apterous viviparous females, Jiuwandashan Mountain (E116.19°, N25.20°), altitude 1000 m, 2.viii.2003, on a kind of fern, by J.Y. Yang (No. 14652); **Guangdong** : 2 apterous viviparous females, Ruyuan County (E113.21°, N24.09°), altitude 1318 m, 18.vii.2008, on *Cyclosorus dentatus?* by X.M. Su (No. 21855); **Hainan** : 5 apterous viviparous females, Changjiang Lizu Autonomous County (E109.18°, N19.09°), altitude 1015 m, 8.v.2007, on a kind of fern, by D. Zhang (No. 19610); **Sichuan** : 1 apterous viviparous female, Chengdu City (E103.07°, N31.05°), altitude 722 m, 12.v.2009, on a kind of fern, by X.M. Su (No. 22519); **Guizhou** : 5 apterous viviparous females, Mayang River (E108.5°, N28.34°), altitude 537 m, 11.vi.2007, on a kind of fern, by Y. Fang (No.19749); 4 apterous viviparous females, Zunyi City (E107.22°, N28.22°), altitude 854 m, 3.vi.2010, on *Cyclosorus acuminatus,* by X.M. Su (Nos. 24511, 24519); 2 apterous viviparous females, Zunyi City (E107.22°, N28.22°), altitude 854 m, 4.vi.2010, on *Cyclosorus acuminatus,* by X.M. Su (No. 24534); 2 apterous viviparous females, Zunyi City (E107.09°, N28.13°), altitude 1534 m, 5.vi.2010, on *Cyclosorus acuminatus,* by X.M. Su (No. 24550); 1 alate viviparous female, 3 apterous nymphs, Zunyi City (E107.22°, N28.22°), altitude 854 m, 11.viii.2010, on *Cyclosorus acuminatus,* by X.M. Su (Nos. 25595, 25597, 25581, 25600); 2 apteous viviparous females, Zunyi City (E107.22°, N28.22°), altitude 854 m, 12.viii.2010, on *Cyclosorus acuminatus,* by X.M. Su (No. 25602); 4 apteous viviparous females, Zunyi City (E107.22°, N28.22°), altitude 854 m, 13.viii.2010, on *Cyclosorus acuminatus,* by X.M. Su (Nos. 25614, 25617); 2 apteous viviparous females, Zunyi City (E107.1°, N28.2°), altitude 1470 m, 16.viii.2010, on *Cyclosorus acuminatus,* by X.M. Su (No. 25636).

***Host plants** : Asplenium esculentum, Asplenium* sp. (Aspleniaceae) ( Raychaudhuri 1980 ); *Dryopteris arida* (Aspidiaceae) ( Ghosh 1974a ; Miyazaki 1968 , 1971; Takahashi 1931 ; Tao 1963 , 1964); *Cheilanthes farinosa* (Pteridaceae) ( Ghosh 1964 ); *Diplazium esculentum* (Aspleniaceae) ( Ghosh 1973, 1977 ; Raychaudhuri 1973 , 1980); *Diplazium japonicum* (Aspleniaceae), *Polystichum rigens* ? (Blackman and Eastop 2006); *Polystichum* sp. ( Tao 1963 ); *Pteridium* spp. (Hypolepidaceae) ( Paik 1972 ). *Cyclosorus acuminatus* and *C. dentatus* ? (Thelypteridaceae) are newly recorded to the list of the host plants ( Figure 17D ).

***Distribution*** : China (Fujian, Guangdong, Hainan, Guangxi, Guizhou, Hubei, Sichuan, Taiwan), Japan, Korea, India, Indonesia, Malaysia, New Britain, Thailand, Philippines and Indonesia (Sumatra).

***Biology*** : The species infests on the undersides of fronds of ferns ( Figure 17D ).

*Macromyzus* **Takahashi**

*Macromyzus* Takahashi, 1960 : 225. Type species: *Myzus woodwardiae*Takahashi, 1921

***Comment*** : Genus *Macromyzus* is divided into two subgenera, *Macromyzus* and *Anthracosiphoniella* , and is restricted to East Asia, including five species mainly colonizing on ferns. In China, the genus is represented by three species belonging to two subgenera, namely, *M* . ( *Anthracosiphoniella* ) *maculatus* , *M* . ( *Macromyzus* ) *spinosus* and *M* . ( *Macromyzus* ) *woodwardiae* .

**Keys to species of** *Macromyzus* **in China** *Apterous viviparous female*

1. Antennal segment III always with secondary rhinaria; thoracic nota and abdominal tergites II–V with pairs of spinal sclerites, and dorsum of body without reticulations and hairbearing tubercles ( Figure 1e )..….… *Macromyzus* ( *Anthracosiphoniella* ) *maculatum*

- Antennal segment III without secondary rhinaria; dorsum of body with distinct reticulations or hairbearing tubercles, or sclerites………………………………….2

2. Antennal setae very short with blunt apices, 0.36–0.46 times as long as basal diameter of antennal segment III; dorsal setae of body with dark sclerites at bases, which with dense ornaments and big spinules ( Figure 1f ); processus terminalis 6.08–6.43 times as long as the base of segment VI……………………………….. ……. *Macromyzus* ( *Macromyzus* ) *spinosus*

-Antennal setae long with blunt apices,1.00–1.30 times as long as basal diameterof the segment; dorsal setae of body withdark brown sclerites at bases, which without any ornaments, or sometimes onlywith very few big spinules ( Figure 1g );processus terminalis 5.196.31 times as long as the base of segment VI…………...……………… *Macromyzus* ( *Macromyzus* )

*woodwardiae*

*Alate viviparous female*

(Apterae of *Macromyzus spinosus* Qiao & Su absent)

1. Antennal segments III-IV with 16–19 and 3–9 secondary rhinaria, respectively; processus terminalis 5.79–6.92 times as long as base of the segment; ultimate rostral segment 1.81–2.03 times as long as second hind tarsal segment………………… ………………. *Macromyzus woodwardiae*

-Antennal segments III-IV with 14–28 and1–6 secondary rhinaria, respectively; processus terminalis 4.82–5.44 times as longas base of the segment; ultimate rostralsegment 1.33–1.55 times as long as second hind tarsal segment………………………… *Macromyzus* ( *Anthracosiphoniella* )

*maculatum*

**Figure 7:**
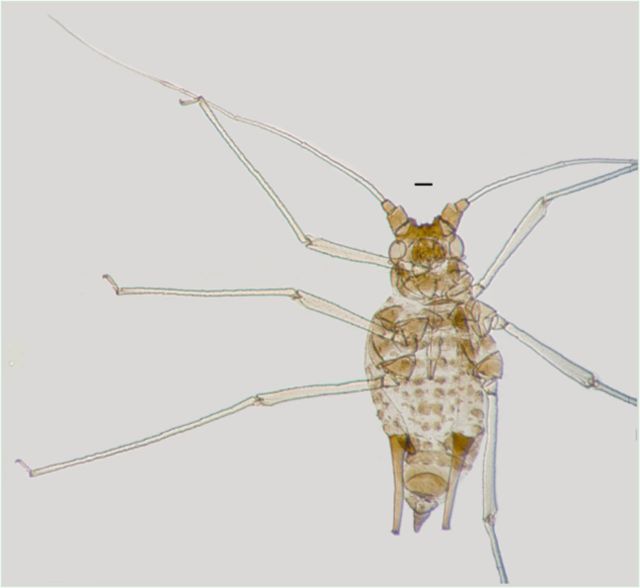
Apterous viviparous female of *Macromyzus spinosus* . Scale bar = 0.10 mm.

***Macromyzus spinosus* Qiao & Su** ( Figure 7 ) *Macromyzus spinosus* Qiao & Su, 2010: 1.

***Material examined** :* **China. Hunan** : 2 apterous viviparous females and 1 apterous nymph (Holotype and paratypes), Yizhang County (E112.09°, N24.10°), altitude 1271 m, 14.vii.2008, on *Plagiogyria japonica,* X.M. Su (No. 21804); 2 apterous viviparous females, Hunan, Yizhang County (E112.09°, N24.10°), altitude 1271 m, 14.vii.2008, on *Plagiogyria japonica* (Plagiogyriaceae), X.M. Su (No. 21808).

***Host plant** : Plagiogyria japonica* (Plagiogyriaceae).

***Distribution** :* China (Hunan).

***Biology** :* The species infests the undersides of fronds of fern, without causing any deformations to the hosts, and was not attended by ants.

*Macromyzus woodwardiae* **(Takahashi)**

( Figure 8 )

**Figure 8.**
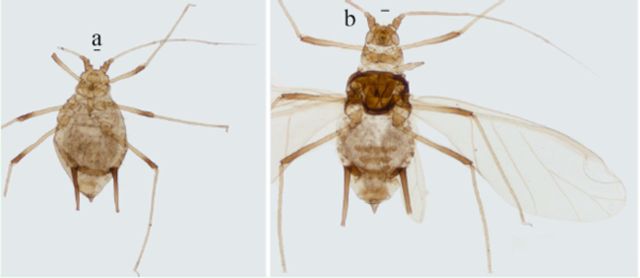
*Macromyzus woodwardiae* . **a.** apterous viviparous female. **b.** alate viviparous female. Scale bars = 0.10 mm.

*Myzus woodwardiae* Takahashi, 1921 : 20. *Macrosiphum woodwardiae*Takahashi, 1925b : 3.

*Myzus woodwardiae hinoi* Moritsu, 1952 : 26. *Macromyzus woodwardiae* (Takahashi): Takahashi, 1960 : 225; Tao, 1963 : 168; 1990: 278; 1999: 73; Miyazaki, 1968 : 19; 1971: 81; 1972: 36; Eastop & Hille Ris Lambers, 1976 : 248; Raychaudhuri, 1980 : 165; Remaudière & Remaudière, 1997 : 108; Lee, 2002 : 127; Blackman & Eastop, 2006: 1191.

***Material examined** :* **China. Hainan** : 4 apterous viviparous females, Ledong County (E109. 02°, N18.44°), altitude 156 m, 24.iii.1984, on a kind of fern, by T.S. Zhong and G.X. Zhang (No. 7855); **Chongqing** : 2 apterous viviparous females and 9 apterous nymphs, Jingyunshan Mountain (E106.55°, N29.55°), altitude 275 m, 27.iv.1991, on a

kind of fern, by T.S. Zhong and G.X. Zhang (No. 10014); **Hunan** : 14 apterous viviparous females, Guidong County (E113.07°, N25.10°), altitude 1143 m, 10.vii.2008, on *Woodwardia japonica,* by X.M. Su (Nos. 21757, 21774, 21778); 2 apterous viviparous females and 1 apterous nymph, Guidong County (E113.08°, N26.07°), altitude 1525 m, 12.vii.2008, on *Woodwardia japonica,* by X.M. Su (No. 21801); 1 apterous viviparous female, Yizhang County (E112.09°, N24.09°), altitude 1271 m, 14.vii.2008, on *Woodwardia japonica,* by X.M. Su (No. 21806); 2 apterous viviparous females and 4 alate viviparous females, Yizhang County (E113.16°, N24.10°), altitude 1339 m, 17.vii.2008, on *Woodwardia japonica,* by X.M. Su (Nos. 21840, 21842); **Guangdong** : 14 apterous viviparous females and 7 alate viviparous females, Ruyuan County (E113.02°, N24.93°), altitude 1021 m, 16.vii.2008, on *Woodwardia japonica,* by X.M. Su (Nos. 21818, 21819, 21820, 21821, 21826, 21829, 21830, 21833, 21834); 2 apterous viviparous females, Ruyuan County (E113.03°, N24.94°), altitude 1318 m, 18.vii.2008, on *Woodwardia japonica,* by X.M. Su (Nos. 21858, 21871); 16 apterous viviparous females and 5 alate viviparous females, Ruyuan County (E113.03°, N24.94°), altitude 1394 m, 19.vii.2008, on *Woodwardia japonica,* X.M. Su (Nos. 21860, 21861, 21862, 21863, 21864, 21870, 21874, 21877, 21893); 1 apterous viviparous female and 4 alate viviparous females, Ruyuan County (E113.02°, N24.94°), altitude 1266 m, 20.vii.2008, on *Woodwardia japonica,* by X.M. Su (No. 21889); 1 apterous viviparous female, Ruyuan County, (E113.02°, N24.94°), altitude 1266 m, 20.vii.2008, on *Arachniodes chinensis,* by X. M. Su (No. 21878); 1 apterous viviparous female, Ruyuan County (E113.02°, N24.94°), altitude 1266 m, 20.vii.2008, on *Diplopterygium glaucum,* by X.M. Su (No. 21880); 4 apterous viviparous females, Ruyuan County (E113.03°, N24.92°), altitude 831 m, 21.vii.2008, on *Blechnum oritental,* by X.M. Su (No. 21902); 6 apterous viviparous females, Ruyuan County (E113.03°, N24.92°), altitude 831 m, 21.vii.2008, on *Woodwardia japonica,* by X.M. Su (Nos. 21901, 21903, 21914); 17 apterous viviparous females and 2 alate viviparous females, Shixing County (E114.21°, N24.74°), altitude 508 m, 23.vii.2008, on *Woodwardia japonica,* by X.M. Su (Nos. 21918, 21920, 21924, 21928, 21931, 21923, 21933, 21935, 21937); 1 apterous viviparous female, Shixing County (E114.21°, N24.74°), altitude 508 m, 23.vii.2008, on *Dryopteris fuscipes,* by X.M. Su (No. 21919); 1 apterous viviparous female, Shixing County (E114.26°, N24.72°), altitude 590 m, 23.vii.2008, on *Microlepia margniata,* X.M. Su (No. 21936); 2 apterous viviparous females and 2 alate viviparous females, Shixing County (E114.21°, N24.74°), altitude 508 m, 23.vii.2008, on *Dryopteris fuscipes,* by X.M. Su (No. 21926); 4 apterous viviparous females, Shixing County (E114.28°, N24.72°), altitude 522 m, 24.vii.2008, on *Woodwardia japonica,* by X.M. Su (Nos. 21951. 21962).

***Distribution** :* China (Chongqing, Hunan, Guangdong, Hainan, Guangxi, Taiwan), India, Indonesia, Japan, Korea and Nepal.

***Host plants** :* Primary hosts: *Hydrangea invo-lucrata* (Hydrangeaceae) ( Miyazaki 1968 ; Moritsu 1983 ); *H. macrophylla* ( Miyazaki 1972 ); *H. macrophylla yezoensis, H. panicu-lata, H. scandens, H. sikokiana,* (Hydrangeaceae) ( Moritsu 1983 ). Secondary hosts: *Asplenium adiantum-nigum* (Aspleniaceae), *A. auriculatum* (Aspleniaceae), *Athyrium macrocarpum* (Athyriaceae), *Diche-ria alata* (Polypodiaceae), *Diplazium esculentum* (Aspleniaceae), *Niphrodium moli,polypodium* sp. (Polypodiaceae), *Thelypterisdentata* (Thelypteridaceae) ( Raychaudhuri 1980 ); *Asplenium cunicularium* , *A. esculentum* (Aspleniaceae) ( Ghosh 1974a ); *Cyrtomium falcatum* , *Cyrtomium presl* ( Lee2002 ); *Dryopteris monticola* (Dryopteridaceae), *Dryopteris varia* (Dryopteridaceae), *Rumobra mutica* ( Miyazaki 1968 , 1971); *Woodwardia* sp.(Blechnaceae), *Polystichum* sp. (Dryopteridaceae) ( Takahashi 1921 ; Tao 1963 ); *Athyrium macrocarpus* , *Woodwardiaradicans* (Blackman and Eastop 2006); *Diplazium esculentum* (Aspleniaceae) (Ghosh

1974a, 1977; Ghosh, Ghosh and Raychaudhuri 1970 ; Raychaudhuri 1973 ); *Asplenium cunicularium* (Aspleniaceae)(Ghosh 1974); *Rumohra mutica* (Aspidiaceae)( Ghosh 1974a ; Higuchi and Miyazaki 1969 ;Miyazak, 1968, 1971; Moritsu 1983 ); *Deutziagracilis* (Hydrangeaceae) ( Moritsu 1983 ); *Dryopteris monticola* (Aspidiaceae) ( Higuchiand Miyazaki 1969 ; Miyazaki 1968 , 1971; Moritsu 1983 ); *Arachniodes chinensis* (Dryopteridaceae), *Blechnumoritentale* (Blechnaceae), *Diplopterygium glaucum* (Gleicheniaceae), *Dryopteris fuscipes* (Dryopteridaceae), *Microlepia marginata* (Dennstaedtiaceae) and *Woodwardia japonica* (Blechnaceae).

***Biology*** : The species colonized on undersides or upsides of fronds and new growth of ferns ( Figure 17G ) in many genera. Anholocyclic on ferns in most places, but in Japan, it is apparently also partially heteroecious holocyclic. Adult fundatrices occurred in late April or early May. They infest young leaves and tender shoots of *Hydrangea* spp., without causing any deformations to the hosts ( Miyazaki 1972 ).

**Figure 9.**
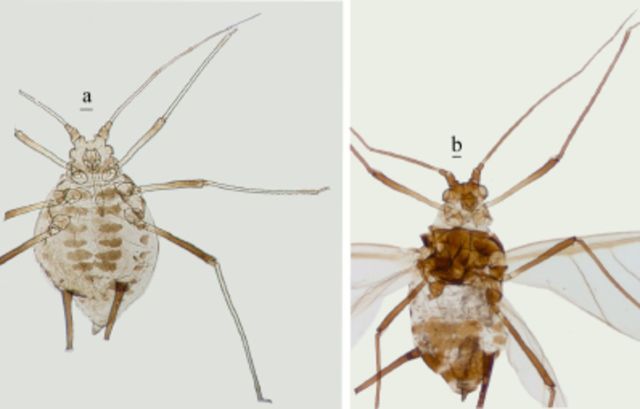
*Macromyzus maculatus* . **a** . apterous viviparous female. **b** . alate viviparous female. Scale bars = 0.10 mm.

*Macromyzus (Anthracosiphoniella) maculatus* **(Basu)** ( Figure 9 )

*Anthracosiphoniella maculatus* Basu, 1969 : 169; Eastop & Hille Ris Lambers, 1976 : 32. *Macromyzus maculatus* (Basu): Raychaudhuri, 1980: 166; Remaudière & Remaudière, 1997 : 108; Blackman & Eastop, 2006: 1191; Su & Qiao, 2010: 1.

***Material examined** :* **China. Sichuan** : 7 apterous viviparous females and 5 alate viviparous females, Emei Mountain, (E103.32°, N29.32°), 12.viii.1936, on a kind of fern, by Takahashi, R. (No. Y7957); **Guangxi** : 1 apterous viviparous female and 1 apterous nymph, Napo County (E105.82°, N23.38°), altitude 924 m, 4.iv.1998, on a kind of fern, by G.X. Qiao (No. 11733); **Tibet** : 4 apterous viviparous females, Pailong County, Yarlung Tsangpo Grand Canyon (E95.00°, N30.02°), altitude 2086 m, 2.ix.2005, on a kind of fern, by D. Zhang (No. 18393); **Guizhou** : 4 apterous viviparous females, Zunyi City (E107.22°, N28.22°), altitude 854 m, 3.vi.2010, on *Phegopteris decursive-pinnata,* by X.M. Su (Nos. 24518, 24520); 1 alate viviparous female, Zunyi City (E107.22°, N28.22°), altitude 854 m, 3.vi.2010, on *Pronephrium penangianum,* by X.M. Su (No. 24521); 6 apterous viviparous females and 2 alate viviparous females, Zunyi City (E107.22°, N28.22°), altitude 854 m, 4.vi.2010, on *Athyriopsis ptersenii,* by X.M. Su (Nos. 24527, 245312, 24532, 24536); 4 apterous viviparous females and 1 alate viviparous female, Zunyi City (E114.28°, N28.13°), altitude 1534 m, 6.vi.2010, on *Athyriopsis ptersenii,* by X.M. Su (Nos. 24552, 24555, 24556); 1 alate viviparous female, Zunyi City (E114.28°, N28.13°), altitude 1534 m, 7.vi.2010, *on Athyriopsis ptersenii,* by X.M. Su (No. 24559); 1 apterous viviparous female, Zunyi City (E107.15°, N28.21°), altitude 1369 m, 8.vi.2010, on *Athyriopsis ptersenii* , by X.M. Su (No. 24579); 1 alate viviparous female and 1 apterous viviparous female, Zunyi City (E107.15°, N28.21°), altitude 1369 m, 9.vi.2010, on *Parathelypteris nipponica* , by X.M. Su (No. 24587); 2 apterous viviparous females, Zunyi City (E107.22°, N28.22°), altitude 854 m, 13.viii.2010, on *Athyriopsis ptersenii* , by X.M. Su (Nos. 25608, 25624); 3 apterous viviparous females, 1 apterous nymph, Zunyi City (E114.28°, N28.13°), altitude 1534 m, 14.viii.2010, on *Parathelypteris glanduligera* ? by X.M. Su (No. 25625); 3 apterous viviparous females, Zunyi City (E114.28°, N28.13°), altitude 1534 m, 15.viii.2010, on *Pronephrium penangianum* , by X.M. Su (Nos. 25630, 25633).

***Host plants*** : *Dryopteris molli* (Aspidaceae), *Asplenium esculentum* (Aspleniaceae), *Athyrium* sp. (Polypodiaceae), *Diplazium esculentum* (Aspleniaceae), *Thelypteris dentata* (Thelypteridaceae) ( Basu, 1969 ; Ghosh, 1974a , 1977; Raychaudhuri, 1973 , 1980); *Eriosorus* sp. (Blackman and Eastop, 2006); *Athyriopsis ptersenii* (Athyriaceae), *Parathelypteris glanduligera* ? (Thelypteridaceae), *Phegopteris decursive-pinnata* (Thelypteridaceae) and *Pronephrium penangianum* (Thelypteridaceae).

***Distribution*** : China (Guangxi, Guizhou, Sichuan, Tibet) and India.

***Biology*** : The species infests on the undersides of fronds of ferns ( Figure 17E ). Apparently anholocyclic ( Ghosh 1974a ).

*Micromyzella* **Eastop**

*Micromyzella* Eastop, 1955 : 203. Type species: *Myzus pterisoides*Theobald, 1918 .

***Comment*** : The genus was originally described as subgenus of *Micromyzus* van der Goot, but with 2-3 setae on first tarsal segments, wing veins normal, and alatae with a dorsal spinal patch. The genus is composed of 12 mostly fern-feeding aphids, and only one species distributes in China.

*Micromyzella judenkoi* **(Carver)**

*Micromyzus judenkoi* Carver, 1965 : 114; Eastop & Hille Ris Lambers, 1976 : 281; Raychaudhuri, 1980: 204; Tao, 1999 : 80. *Micromyzella judenkoi* (Carver): Remaudière, Autrique, Eastop, Stary & Aymonin, 1985: 175; Remaudière & Remaudière, 1997 : 121; Qiao & Jiang, 2005 : 584; Blackman & Eastop, 2006: 1229.

***Material examined*** : **China. Hong Kong** : 2 alate viviparous females, E114.1°, N22.38°, altitude 460 m, April 1976, trapped in yellow trays, by H.Y. Lee (BMNH); 1 alate viviparous female, E114.1°, N22.38°, altitude 460 m, April 1975, trapped in yellow trays, H.Y. Lee (BMNH).

***Host plants*** : *Adiantum caudatum* (Adiantaceae) ( Chakrabarti 1984 ); *Asplenium* (Aspleniaceae), *Athyrium* sp. (Athyriaceae), *Cheilanthes* sp., *Cheilanthes compositor* (Pteridaceae) ( Ghosh, Ghosh and Raychaudhuri 1970 ; Ghosh 1974a ; Raychaudhuri 1973 , 1980).

***Distribution*** : China (Hong Kong), Australia, India, Philippines, Sri Lanka and Thailand. ***Biology*** : The species infests the undersides of fronds of ferns.

*Micromyzodium* **David**

*Micromyzodium* David, 1958 : 175. Type species: *Micromyzodium filicium*David, 1958 . *Eomyzus*Takahashi, 1960 : 227. Type species: *Myzus nipponicus*Moritsu, 1949 .

***Comment*** : The genus is related to *Micromyzus* and *Micromyzella* , but members of *Micromyzodium* have long dorsal body setae and are mainly distributed in Asia. Two species colonized ferns worldwide, along with one species distributed in China.

**Figure 10.**
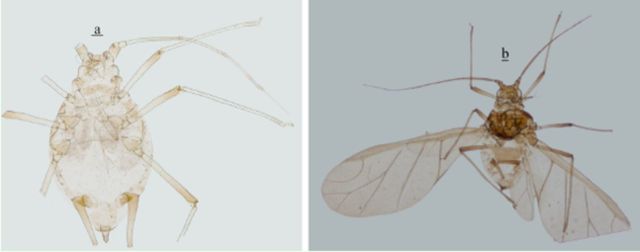
*Micromyzodium polypodii* . **a.** apterous viviparous female. **b** . alate viviparous female. Scale bars = 0.10 mm.

*Micromyzodium polypodii* **Takahashi** (new record for China) ( Figure 10 )

*Micromyzodium polypodii* Takahashi, 1963 : 61.

*Micromyzodium polypodii* Takahashi: Miyazaki, 1971: 79; Remaudière & Remaudière, 1997 : 121; Blackman & Eastop, 2006: 1230.

***Material examined*** : **China. Jiangxi** : 2 apterous viviparous females, Jinggangshan City,(E114.13°, N26.53°), altitude 909 m,8.ix.1995, on a kind of fern, by G.X. Zhang(No. 10854); **Hunan** : 7 alate viviparous females and 3 apterous viviparous females, CiliCounty (E111.13°, N29.4°), altitude 131 m,17.x.1988, on a kind of fern, by T.S. Zhongand W.Y. Zhang (Nos. 8984, 8986); **Guizhou** :6 apterous viviparous females, LeigongshanMountain (E108.28°, N26.47°), altitude 1630m, 31.v.2005, on a kind offern, by Y. Fang(No. 16204); **Fujian** : 4 apterous viviparousfemales, Wuyishan Mountain (E109.48°,N30.27°), altitude 461 m, 20.x.2005, on a kindof fern, by L.Y. Jiang (No. 18044); **Zhejiang** :4 apterous viviparous females, Fengyangshan Mountain (E109.33°, N27.08°), altitude 491m, 28.vii.2007, on a kind of fern, by Y. Fang(No. 20391); 5 apterous viviparousfemales,FengyangshanMountain(E109.33°,N27.08°), altitude 491 m, 1.viii.2007, on a kind of fern, by Y. Fang (No. 20432); **Hunan** : 1 alate viviparous female and 6 apterous viviparous females, Guidong County (E113.12°, N25.17°), altitude 1143 m, 10.vii.2008, on *Parathelypteris glanduligera* , by X.M. Su (Nos. 21761, 21764); **Fujian** : 2 apterous viviparous females, Nanjing County (E117.22°, N24.08°), altitude 332 m, 21.xi.2008, on a kind of fern, H.H. Zhang (No. 22141).

***Host plants*** : *Parathelypteris glanduligera* (Thelypteridaceae), and unidentified ferns in Japan ( Miyazaki 1971 ).

***Distribution*** : China (Fujian, Guizhou, Hunan, Jiangxi, Zhejiang) and Japan.

***Biology*** : Colonies are formed on the undersides of fronds of ferns.

*Micromyzus* **van der Goot**

*Micromyzus* van der Goot, 1917 : 52. Typespecies: *Micromyzus nigrum*van der Goot, 1917 .

*Myzopsis* Matsumura, 1918 : 19. Type species: *Myzopsis diervillae*Matsumura, 1918 .

***Comment*** : Ten species infesting fern distribute in Eastern Asia. The genus is closed to *Micromyzella* , but first tarsal segments with 4 setae, and alatae having dark-bordered wing veins and a strongly curved radial sector. Only one species is reported in China.

**Figure 11.**
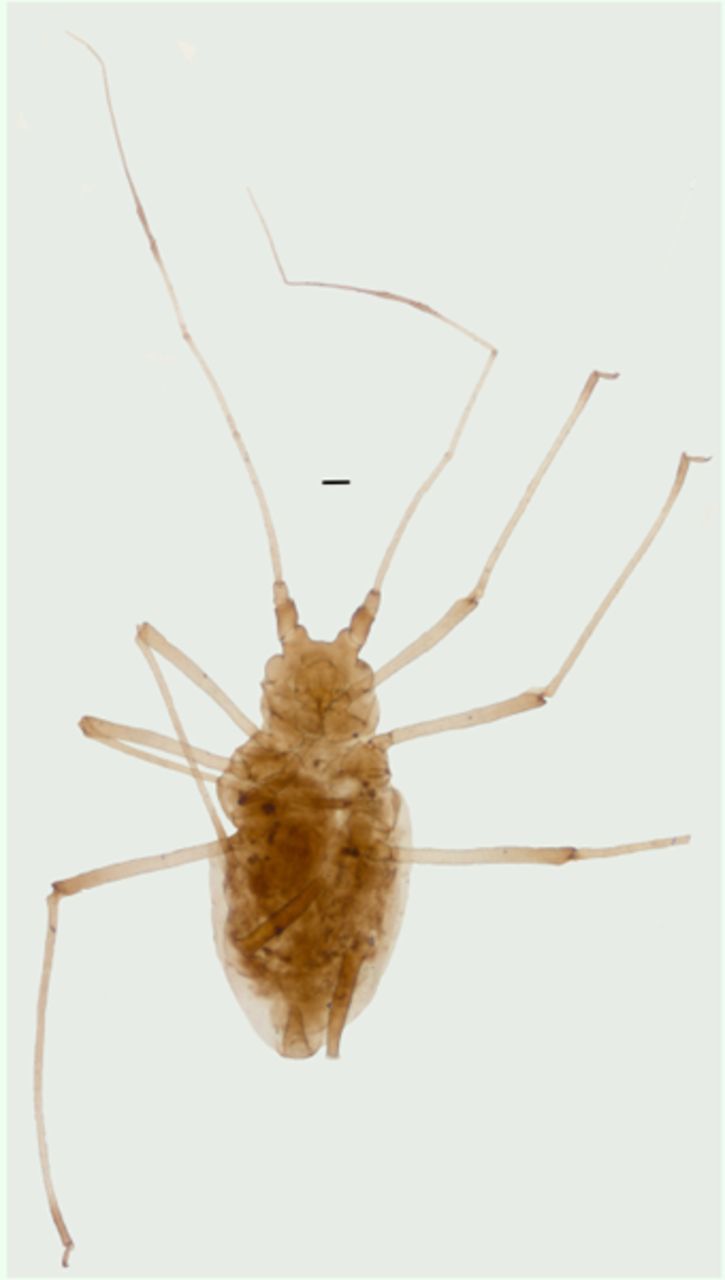
Apterous viviparous female of *Micromyzus kaoti* . Scale bar = 0.10 mm.

*Micromyzus katoi* **(Takahashi)** ( Figure 11 )

*Amphorophora katoi* Takahashi, 1925b : 25. *Micromyzus katoi* (Takahashi): Eastop & Hille Ris Lambers, 1976 : 281; Tao, 1999 : 80; Remaudière & Remaudière, 1997 : 122; Noordam 2004 : 101; Blackman & Eastop, 2006: 1230.

***Material examined*** : **China. Taiwan** : 7 apterous viviparous females and 5 apterous nymphs, E120.95°, N23.7°, altitude 1090 m, 15.xi.1924, on a kind of fern, by R. Toyota (No. Y7950).

***Host plants*** : *Polypodium ellipticum* ( Takahashi 1925a , 1931; Tao 1963 , 1967), *Platycerium* sp. (Platyceriaceae), *Polypodium punctatum* ( Noordam 2004 ); *Microsorium* sp. (Polypodiaceae) (Blackman and Eastop 2006). ***Distribution*** : China (Taiwan), Indonesia and Australia.

***Biology*** : The aphids infest the lower sides of fronds of ferns.

*Myzus* **Passerini**

*Myzus* Passerini, 1860 : 27. Type species: *Aphis cerasi*Fabricius, 1775 .

*Prunomyzus* Hille Ris Lambers & Rogerson,1946 : 105. Type species: *Myzus* ( *Prunomyzus* )

*padellus* Hille Ris Lambers & Rogerson,1946 .

***Comment*** : In the genus, only a species of fern-feeding aphid, *Myzus filicis* Basu, was discovered on an unidentified fern from India and Nepal. The species is distributed in China, too.

**Figure 12.**
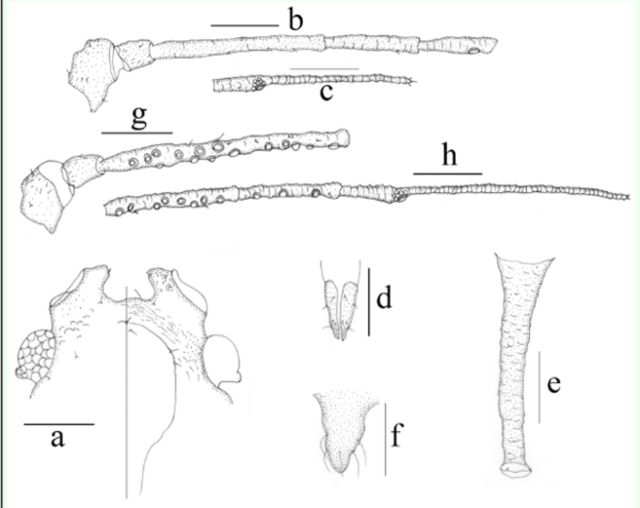
*Myzus filicis* . ( **a–f** ): apterous viviparous female: **a.** dorsal(left) and ventral (right) views of head. **b** . antennal segments I–V. **c** .antennal segment VI. **d** . ultimate rostral segment. **e** . siphunculus. **f.** cauda. ( **g–h** ). alate viviparous female: **g** . antennal segments I–III. **h** . antennalsegments IV–VI. Scale bars = 0.10 mm.

**Figure 13.**
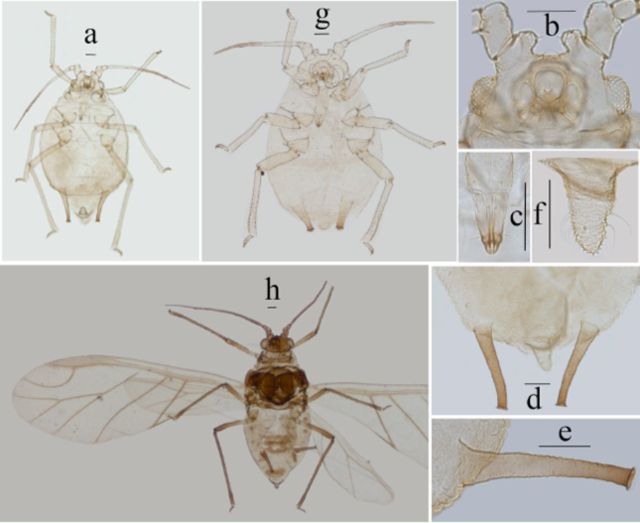
*Myzus filicis* . ( **a–f** ): apterous viviparous female: **a.** dorsalview of body. **b.** dorsal view of head. **c.** ultimate rostral segment. **d** .abdominal tergites IV–VIII (C-, or O-shaped wrinkles on dorsum ofbody shown). **e.** siphunculus. **f** . cauda. **g** . dorsal view of body of apterousnymph. **h** . dorsal view of body of alate viviparous female. Scale bars= 0.10 mm.

***Myzus filicis*** **Basu** (new record for China) ( Table 2 , Figures 12–13 )

**Table 2.**
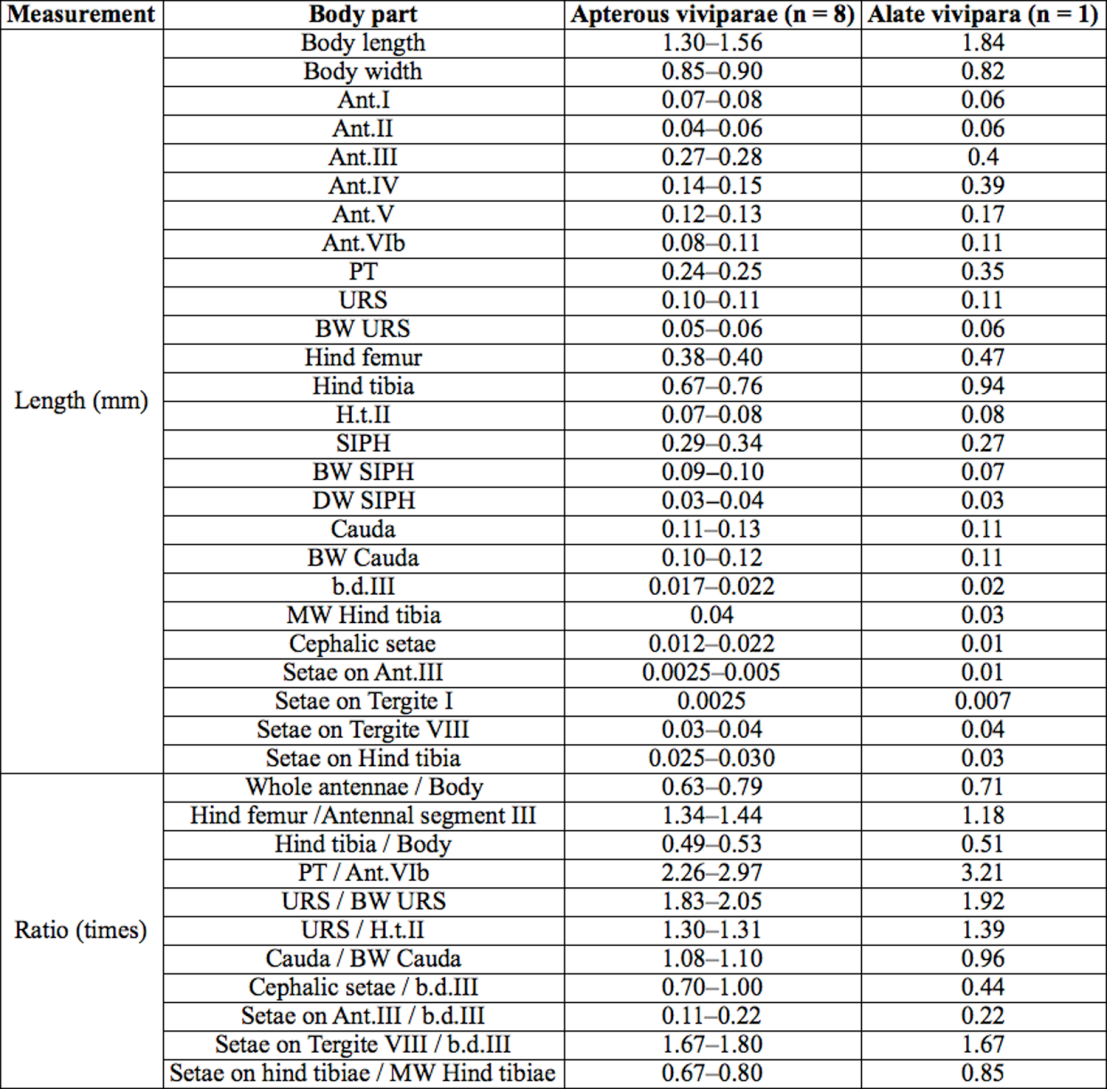
Biometric data (mean, range) of *Myzus filicis* Basu (in mm).

*Myzus filicis* Basu, 1969 : 181. *Myzus filicis* Basu: Eastop & Hille Ris Lambers, 1976 : 297; Raychaudhuri, 1980 : 213; Remaudière & Remaudière, 1997 : 125; Blackman & Eastop, 2006: 1239.

Basu (1969) gave a concise description of apterae. On the basis of the present specimens,redescription of apterae and description of the hitherto unknown alatae are given below.

***Apterous viviparous female** :* Body roundly oval, yellow in life.

*Mounted specimens:* body pale ( Figure 13a ) and broad oval, 1.30-1.56 mm long and 0.85-0.90 mm wide. General measurements see Table 2 .

*Head:* slightly brown; with sparse wrinkles near eyes dorsally and spinulose ventrally ( Figures 12a , 13b ). Middle frontal tubercle indistinct, antennal tubercles very developed, with spinulose imbrications, converging at inner sides, with 3 pairs of antennal tubercles setae and a pair of median frontal setae. Dorsal setae of head short and slightly blunt apices, with 2 pairs of setae between antennae, arranged longitudinally and the anterior setae a little longer than the posterior ones; 2 pairs of setae between compound eyes, arranged transversely. Cephalic setae 0.012-0.022 mm long, the anterior dorsal setae about as long as cephalic ones, the posterior ones 0.0025 mm long, 0.70-1.00 and 0.10-0.11 times as long as basal diameter of antennal segment III, respectively. Ventral setae slightly longer than dorsal ones. Eyes with distinct ocular tubercles. Antennae 6-segmented, distal part of antennal segment III and antennal segments IV-VI dark brown, segments I and II smooth except for inner margin of segment I with sparse imbrications, segments III-VI imbricated, ones on segment III weak ( Figures 12b , 12c ). Antennae 0.63-0.79 times as long as body, length in proportion of segments I-VI: 25-27, 16-21, 100, 53-55, 44–46, 33-38+85-94; processus terminalis 2.26-2.97 times as long as basal part of the segment. Antennal setae short and blunt, segments I-VI each with 3 or 4, 3 or 4, 5 or 6, 2, 2, 2+3 setae, respectively, apex of processus terminalis with 3 setae; setae on segment III 0.11-0.22 times as long as basal diameter of the segment. Primary rhinaria ciliated, secondary rhinaria absent. Rostrum reaching between middle and hind coxae; ultimate rostral segment wedge-shaped, apex dark brown ( Figures 12d , 13c ), 1.83-2.05 times as long as its basal width, 1.30-1.31 times as long as second hind tarsal segment, with 6 primary setae and 4 accessory setae.

*Thorax:* thoracic nota with C-shaped wrinkles. Venter with spinulous transverse rows. Legs short, tarsi brown, coxae with spinules ventrally, distal parts of femora with sparse spinulous imbrications, and distal parts of hind tibiae very weakly spinulous to smooth, others smooth. Hind femur 1.34-1.44 times as long as antennal segment III. Hind tibiae 0.49-0.53 times as long as body; setae on hind tibiae short with acuminate apices, 0.67-0.80 times as long as middle diameter of the segment. First tarsal chaetotaxy: 3, 3, 2. Hind tibiae of nymph with spinules.

*Abdomen:* abdominal tergites I-VI with C- or O-shaped wrinkles, posterior areas of siphunculi, tergites VII and VIII with spinules ( Figure 13d ). Venter with spinulous transverse rows. Spiracles nephroid, spiracular plates pale and slightly prominent. Dorsal setae on tergites I-VII very short and blunt, tergite VIII with 4 long setae. Siphunculi cylindrical, wholly brown, widest at base, distinct imbricated and with a row of striate under developed flange ( Figures 12e , 13e ), 0.22-0.26 times as long as body, 3.42-4.00 times as long as its basal width, 2.64 times as long as cauda. Cauda pale brown, coniform, distal half slightly constricted ( Figures 12f , 13f ), with 5 setae. Anal plate pale brown, transverse oval, with 8-10 setae. Genital plate pale brown, broad round with 10 posterior setae and 4 anterior setae.

***Alate viviparous female** :* Mounted specimens: body pale ( Figure 13h ), body 1.84 mm long, 0.82 mm wide. General measurements see Table 2 .

**Figure 14.**
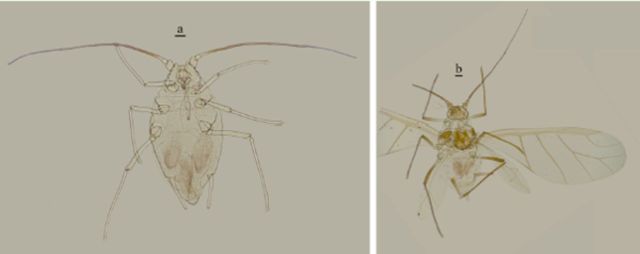
*Shinjia orientalis* . **a** . apterous viviparous female. **b** . alate viviparous female. Scale bars = 0.10 mm.

*Head:* brown, and smooth dorsally and ventrally. Middle frontal tubercle indistinct, antennal tubercles low, parallel at inner sides, with 3 pairs of antennal tubercles setae and a pair of median frontal setae. Dorsal setae of head short and slightly blunt apices, with 2 pairs of setae between antennae, arranged longitudinally; 2 pairs of setae between eyes, arranged transversely. Cephalic setae 0.01 mm long, dorsal setae 0.005 mm long, 0.44 times and 0.22 times as long as basal diameter of antennal segment III, respectively. Antennae 6-segmented, dark brown, segments I and II smooth dorsally except for inner margin of segment I sparse imbricated, segments III-VI with imbrications, ones on segment III weak. Antennae 0.71 times as long as body, length in proportion of segments I-VI: 14, 11, 100 **,** 48, 42, 27+88, processus terminalis 3.21 times as long as the base of the segment. Primary rhinaria ciliated, segments III-V with 18-22, 6-8 and 2 or 3 secondary rhinaria, respectively ( Figures 14g , 14h ). Antennal setae very short and acute, setae on segment III 0.44 times as long as basal diameter of the segment. Rostrum with 6 primary setae and 4 accessory setae.

*Thorax:* legs long, distal parts of femora with sparse imbrications, and distal parts of hind tibiae very weakly spinulous, others smooth. Hind femora 1.18 times as long as antennal segment III. Hind tibiae 0.51 times as long as body; setae on hind tibiae 0.85 times as long as middle diameter of the segment.

*Abdomen:* abdominal tergites I-VI smooth, spinal and lateral patches fused with a large brown patch on abdominal tergites IV-V,tergite VI with a brown stripe and lateral areas connected with the dorsal patch, tergites II-VIIeach with a pair of marginal patches, marginal patches on tergite VI slightly bigger than others, tergite VII with a faint brown band and tergite VIII with a brown spinal ban Posterior areas of siphunculi, tergites VII and

VIIIwith spinulous stripes. tergite VIII with 4setae. Siphunculi cylindrical, brown, distinctimbricated, 0.15 times as long as body, 3.86times as long as its basal width, 2.47 times aslong as cauda. Cauda brown, coniform, distalpart slightly acute, with 5 setae. Anal platetransverse oval, with 10 setae. Genital platebroad round with 10 posterior setae and 4 anterior setae. Wing veins normal. Others are similar to apterae.

***Material exmined** :* **China. Tibet** : 6 apterous viviparous females and 1 alate viviparous female, Zham Town (E86.00°, N28.00°), altitude 2490 m, 28.vii.2005, on a kind of fern, by J.F. Wang (No. 16523); 2 apterous viviparous females, Zham Town (E86.00°, N28.00°), 8.viii.2010, on *Dennstaedtia appendiculata,* by Y.Wang (No. 25803).

***Host plants** : Dennstaedtia appendiculata* (Dennstaedtiaceae), and unidentified ferns (Blackman and Eastop 2006).

***Distribution** :* China (Tibet), India and Nepal.

***Biology** :* Infesting loosely on the undersides of fronds of fern and without causing distinct injury ( Figure 17I ). The species have not been seen visited by ants.

***Comment** :* The specimens from China slightly differ from the Indian specimens: (1) ocular tubercles small but visible (Indian specimens: eye without distinct triommatidia), (2) distal parts of femora with very sparse spinulose imbrication (Indian specimens: femora and tibiae smooth), (3) ultimate rostral segment with 4 accessory setae (Indian specimens: ultimate rostral segment with 2 accessory setae) (the information of Indian specimens from Basu 1969 ).

*Shinjia* **Takahashi**

*Microtarsus* Shinji, 1929 : 43, nec Eyton, 1839. Type species: *Microtarsus pteridifoliae*Shinji, 1929 . *Microtarsus*Shinji, 1930 : 188. Type species: *Microtarsus pteridifoliae*Shinji, 1930*Microtarsus pteridifoliae*Shinji, 1929*Shinjia*Takahashi, 1938 : 6. Type species: *Microtarsus pteridifoliae*Shinji, 1929 .

***Comment** :* The genus is quite distinctive among the Macrosiphini in having reduced tarsi, with first tarsal chaetotaxy 1, 0, 0 and no claws. The genus is represented by a single species feeding on ferns.

*Shinjia orientalis* **(Mordvilko)** ( Figure 14 )

*Microtarsus pterydifoliae* Shinji, 1929 : 44. *Shinjia pterydifoliae* (Shinji): Takahashi, 1938 : 6; Sorin, 1962 : 21; Tao, 1963 : 163; Paik, 1965 : 53; Eastop, 1966 : 475; Miyazaki, 1968 : 21; 1971: 165; Raychaudhuri, 1980 : 250.

*Shinjia orientalis* (Mordvilko): Eastop & Hille Ris Lambers, 1976 : 395; Remaudière & Remaudière, 1997 : 143; Blackman & Eastop, 2006: 1291.

***Material examined** :* **China. Zhejiang** : 5 alate viviparous females, Hangzhou City (E120.15°, N30.27°), altitude 19 m, 20.v.1975, on a kind of fern, by G.X. Zhang (No. 6112); 2 alate viviparous females, Hangzhou City (E120.15°, N30.27°), altitude 19 m, 22.v.1975, on a kind of fern, by G.X. Zhang (No. 5787); 1 alate viviparous female and apterous viviparous females, Fengyangshan Mountain (E109.33°, N27.08°), altitude 491 m, 26.vii.i2007, on a kind of fern, by Y. Fang (No. 20367); **Gansu** : 1 alate viviparous female and 12 apterous viviparous females, Yuzhong County (E104.18°, N350.82°), altitude 1975 m, 30.viii.1986, on a kind of fern, by G.X. Zhang, J.H. Li and T.S. Zhong (No. 8572); **Sichuan** : 2 alate viviparous females and 2 apterous viviparous females, Yajiang City (E101.15°, N30.03°), altitude 3073 m, 27.iv.1991, on a kind of fern, by T.S. Zhong and G.X. Zhang (No. 10001); **Yunnan** : 2 alate viviparous females and 6 apterous viviparous females, Dali Bai Autonomous Region (E100.35°, N25.6°), altitude 2119 m, 27.vi.2005, on a kind of fern, by J.F. Wang (No. 16317); **Tibet** : 3 apterous viviparous females, Zayü County (E97.47°, N28.63°), altitude 1520 m, 12.vii.2005, on a kind of fern, by J.F. Wang (No. 16423); 1 apterous viviparous female, Zayü County (E96.93°, N28.58°), altitude 2014 m, 25.viii.2005, on a kind of fern, by D. Zhang (No. 18342); 2 apterous viviparous females, Zayü County (E97.03°, N28.48°), altitude 1534 m, 26.viii.2005, on a kind of fern, by D. Zhang (No. 18356); 3 apterous viviparous females, Nyingchi Region (E94.47°, N29.55°), altitude 4087 m, 4.viii.2010, on *Pteridium aquilinum* var. *latiusculum,* by Y. Wang (No. 25742); **Hainan** : 4 alate viviparous females and 4 apterous viviparous females, Ledong County (E108.09°, N18.08°), altitude 784 m, 2.v.2007, on a kind of fern, by G.X. Qiao and D. Zhang (No. 19569); **Hunan** : 3 alate viviparous females and 14 apterous viviparous females, Changsha City (E112.93°, N28.22°), altitude 29 m, x.1985, on a kind of fern, by G.X. Zhang (No. 8267); 9 apterous viviparous females, Zhangjiajie City (E110.5°, N29.01°), altitude 217 m, 12.x.1988, on a kind of fern, by T.S. Zhong and W.Y. Zhang (No. 8958); 7 alate viviparous females and 8 apterous viviparous females, Yizhang County (E112.93°, N25.4°), altitude 248 m, 30.x. 1988, on a kind of fern, by T.S. Zhong (No. 9059); 3 apterous viviparous females, Guidong County (E113.07°, N25.10°), altitude 1143 m, 10.vii.2008, on *Pteridium revolutum,* by X.M. Su (No. 21760); 4 apterous viviparous females and 3 alate viviparous females, Guidong County (E113.08°, N25.10°), altitude 1399 m, 11.vii.2008, on *Pteridium revolutum,* by X.M. Su (Nos. 21784, 21788, 21789); 1 alate viviparous female, Guidong County (E113.08°, N26.07°), altitude 1525 m, 12.vii.2008, on *Pteridium revolutum,* by X.M. Su (No. 21796); 2 alate viviparous females and 5 apterous viviparous females, Yizhang County (E113.16°, N24.10°), altitude 1339 m, 17.viii.2008, on *Pteridium revolutum,* by X.M. Su (No. 21844); **Guangdong** : 2 alate viviparous females and 2 apterous viviparous females, Ruyuan County (E113.16°, N24.09°), altitude 1266 m, 20.vii.2008, on *Pteridium revolutum,* by X.M. Su (No. 21888); **Guizhou** : 3 apterous viviparous females, Leigong Mountain (E108.4°, N25.88°), altitude 760 m, 1.vi.2005, on a kind of fern, by Y. Fang (No. 16224); 1 apterous viviparous female, Suiyang County (E107.09°, N28.13°), altitude 1534 m, 7.vi.2010, *on Pteidium aquilinum* var. *latiusculum,* by X.M. Su (No. 24559).

***Host plants** :* Primary hosts: *Viburnum cotin-ifolium* ( Chakrabarti and Banerjee 1993 ); *V. dilatatum, V. erosum, V. japonicum* ( Miyazaki 1968 , 1971; Higuchi and Miyazaki 1969 ; Moritsu 1983 ); *V. sargentii* ( Pashchenko 1988 ); *V. dentatum* (Blackman and Eastop 2006). Secondary hosts: *Artemisia princeps* (Asteraceae) ( Moritsu 1983 ); *Athyrium macrocarpum, Athyrium* sp. (Athyriaceae) (Raychaudhuri 1978, 1980); *Dryopteris* sp. (Aspidiaceae) ( Ghosh 1974b ; Ghosh and Raychaudhuri 1970 ; Raychaudhuri 1973 ); *Lyoniaovalifolia* (Ericaceae) ( Chakrabarti and Sarkar, 2001 ); *Polypodium* sp. (Polypodiaceae) ( Ghosh 1974b ; Raychaudhuri 1973 , 1980); *Pteridium aquilinum* (Hypolepidaceae) ( Ghosh 1974b ; Raychaudhuri 1973 , 1980; Chakrabarti and Raychaudhuri 1975 ; Higuchi and Miyazaki 1969 ; Miyazaki 1968 ; Lee, Holman and Havelka 2002 ); *Pteridium aquilinum japonicum* (Hypolepidaceae) ( Paik 1965 ); *Pteridium aquilinum latiusculum* (Hypolepidaceae) ( Moritsu 1983 ; Paik and Choi 1969 ; Paik, Lee, Woo and Park 1969 ); *Pteris* sp. (Pteridaceae) (Raychaudhuri 1973; Chakrabarti and Sarkar 2001 ; Chakrabarti 1984 , 1985; Chakrabarti, Saha and Mandal 1988 ); *Pteris ovata* (Pteridaceae) ( Chakrabarti and Sarkar 2001 ; Chakrabarti and Raychaudhuri 1975 ). *Pteridium revolutum* is newly added to the list of host plants ( Figure 17F ).

***Distribution*** : China (Guangdong, Guizhou, Hainan, Hunan, Sichuan, Gansu, Yunnan, Tibet, Zhejiang), Japan, Korea, India, Nepal, Russia (East Siberia), Philippines and Australia.

***Biology*** : In spring, the species live on the young growth of *Viburnum* spp. and alter to secondary hosts of ferns in Japan ( Sorin 1962 ). In Australia, it probably is anholocyclic on ferns (Blackman and Eastop 2006).

*Vietaphis* **gen. nov**

***Etymology*** : The genus name consists in “ *vie-tus* (Latin)” (= wrinkled) and “ *aphis* (Latin)” (= aphid). The genus is named after its dorsum of body with C- or O-shaped wrinkles.

*Type species* : *Vietaphis aliquanti***sp. nov.**

*Gender* : Feminine.

***Diagnosis** :* Body small. Head smooth dorsally and sparse spinulose ventrally. Antennal tubercles developed, parallel or slightly diverging at the inner margins. Eyes with small and distinct ocular tubercles. Antennae 6-segmented, shorter than body length; antennal segment III in apterae without secondary rhinaria, but segments III-V in alatae present. Wing veins normal. First tarsal chaetotaxy: 3, 3, 3. Hind tibiae of nymphs without spinules. Dorsum of head near eyes with C-shaped wrinkles, dorsum of abdomen with C or O-shaped wrinkles in apterae; alatae with dark dorsal patch on abdomen. Marginal tubercles on abdomen absent. Siphunculi cylindrical with dense imbrications, no constricted under developed flange. Cauda coniform, distal half slightly constricted with 5 setae.

***Comments** :* The new genus belongs to Macro-shiphini (Aphidinae) in having spiracle on abdominal tergites I and II placed close together, lateral frontal tubercles pronounced, abdominal segments II-V or I and VII without marginal tubercles. Among these genera feeding on ferns in the world, the new genus is distinguished from its allied genera like *Idiopterus* Davis, *Macromyzella* Ghosh, Basu & Raychaudhuri, *Macromyzus* Takahashi, *Micromyzella* Eastop, *Micromyzodium* David and *Micromyzus* van der Goot, by the following characters, such as C- or O-shaped wrinkles on abdominal tergites, antennal segment III of apterae without secondary rhinaria, apex of siphunculi without reticulations, dorsal setae of body short and blunt, and alatae with normal wing veins. The new genus differs from other genera in morphology shown in the key.

Relatively, the new genus is more closely related to *Macromyzella* Ghosh, Basu & Raychaudhuri by the shape of the body and siphunculi compared to other fern-feedingaphids, but differs from *Macromyzella* as follow: (1) dorsum of head with C-shaped wrinkles only near eyes, other areas smooth, abdominal tergites with C- or O-shaped wrinkles ( *Macromyzella* : dorsum of head spinulose, abdominal tergites with reticulations), (2) antennae shorter than body, 0.50– 0.80 times as long as body; processus terminalis short, 1.60–3.00 times as long as basal part of the segment ( *Macromyzella* : antennae as long as or longer than body, 1.00–1.30 times as long as body; processus terminalis long, 4.60–5.80 times as long as basal part of the segment), (3) siphunculi without any reticulations under the flange, with imbrications ( *Macromyzella* : siphunculi with 2-3 lines reticulations under flange, other parts with spinule short transverse stripes).

***Biology*** : Usually, the species of the new genus loosely infest on the distal part of the undersides of fronds, and without causing distinct injury.

**Figure 15.**
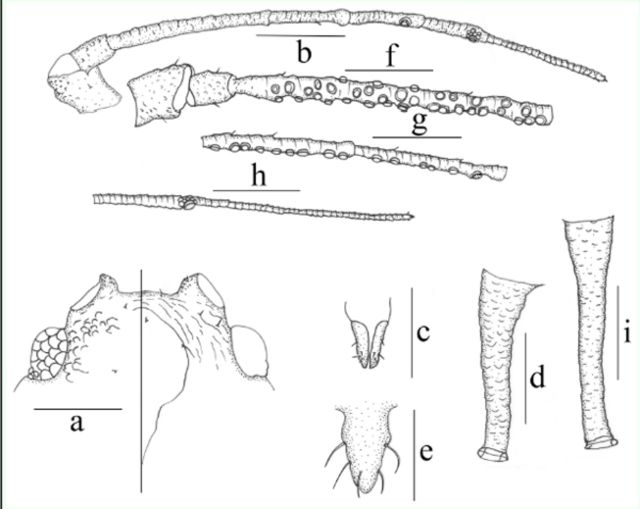
*Vietaphis aliquantus* **sp. nov.** ( **a–e** ) apterous viviparousfemale: **a** . dorsal (left) and ventral (right) views of head. **b** . antennalsegments I-VI. **c** . ultimate rostral segment. **d** . siphunculus. **e** . cauda. **(f–i** ) alate viviparous female: **f** . antennal segments I-III. **g** . antennal segmentsIV-V. **h** . antennal segment VI. **i** . siphunculus. Scale bars = 0.10 mm

**Figure 16.**
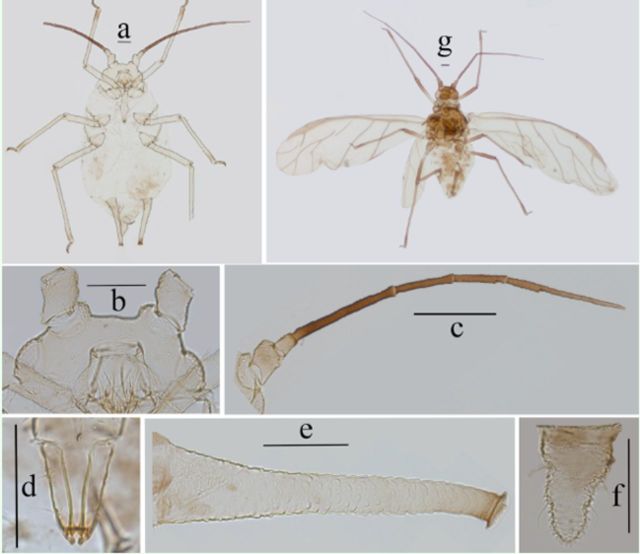
*Vietaphis aliquantus* **sp. nov. (a–f** ) apterous viviparousfemale: **a** . dorsal view of body. **b** . dorsal view of head. **c** . antennal segmentsI–VI. **d** . ultimate rostral segment. **e** . siphunculus. **f** . cauda. **g** .dorsal view of body of alate viviparous female. Scale bars = 0.10 mm.

*Vietaphis aliquantus* **sp. nov.** ( Table 3 , Figures 15–16 )

**Table 3.**
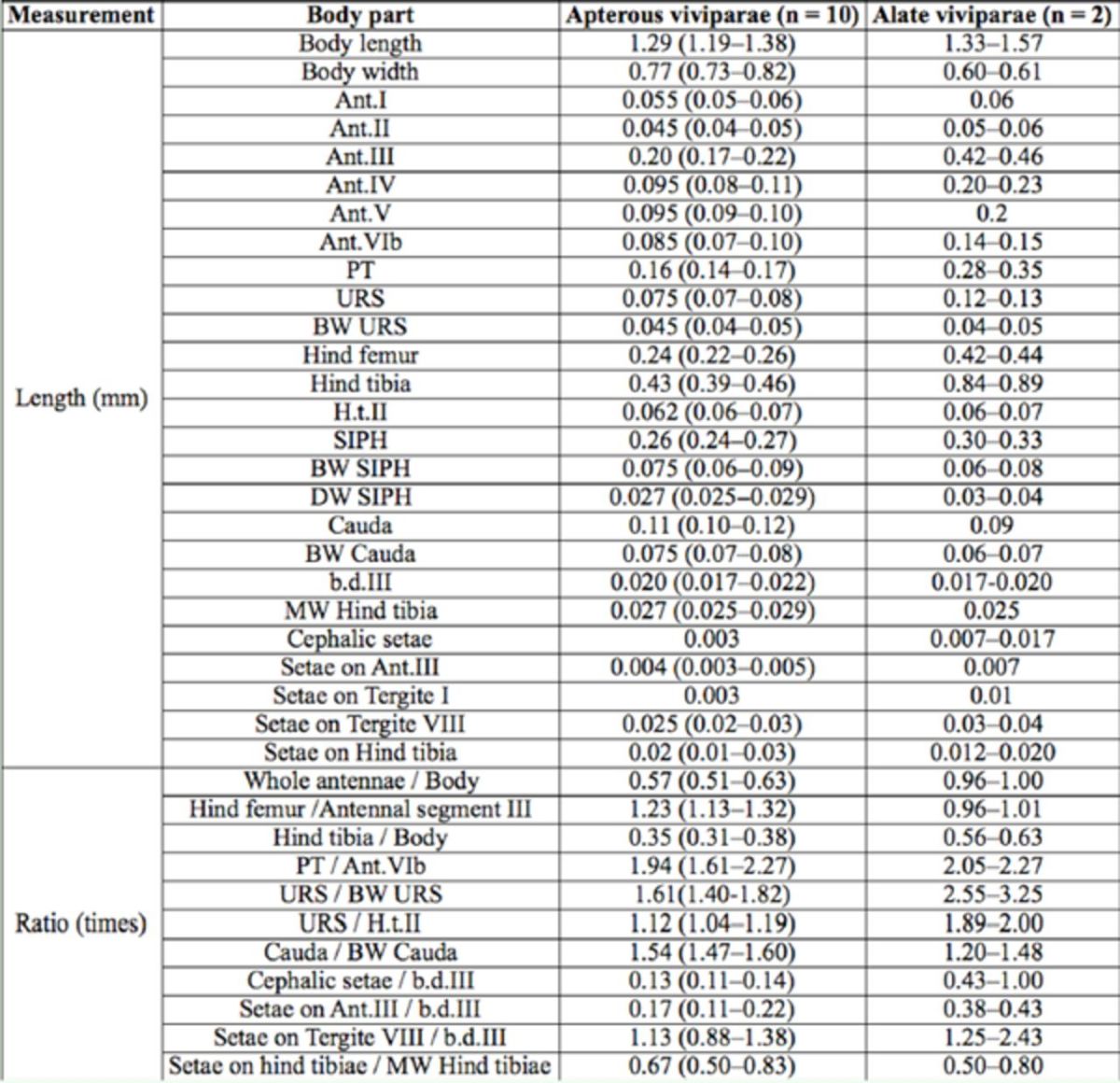
Biometric data (mean, range) of *Vietaphis aliquantus***sp. nov.** (in mm).

***Type locality*** : China (Guizhou (E107.1°, N28.2°, altitude 1470 m), Tibet (E88.9°, N27.48°, altitude 2700 m)).

***Etymology*** : The species name consists in “ *aliquantus* (Latin)” (= moderate). The species is named after its moderately developed antennal tubercles.

***Apterous viviparous female*** . Body oval, yellowish green in life.

*Mounted specimens* : Body pale brown and broad oval ( Figure 16a ). Body 1.19–1.38 mm long, 0.73–0.82 mm wide. General measurements see Table 3 .

*Head:* faint brown. Dorsum of head smooth except for C-shaped wrinkles near eyes dorsally, and sparse spinules and densely wrinkles ventrally ( Figures 15a , 16b ). Middle frontal tubercle indistinct, antennal tubercles moderate developed with imbrications, slightly diverging at inner sides, 2 pairs of antennal tubercles setae and a pair of median frontal setae. Dorsal setae of head extremely short and slightly blunt apices, 2 pairs of setae between antennae, arranged longitudinally; and 2 pairs of setae between eyes, arranged transversely. Cephalic setae about as long as dorsal setae, 0.0025 mm long, 0.11-0.15 times as long as basal diameter of antennal segment III. Ventral setae of head slightly longer than dorsal ones. Eyes big with small ocular tubercles. Antennae 6-segmented, inner margin of segment I, and segments II-VI dark brown and imbricated ( Figures 15b , 16c ). Antennae 0.51-0.63 times as long as body length, length in proportion of segments I-VI: 27-29, 23-24, 100, 47-50, 44–52, 41-45+77-82, segment IV almost as long as segment V, processus terminalis 1.61-2.27 times as long as base of the segment. Antennal setae similar to head dorsal setae, extremely short and blunt, segments I-VI each with 2-4, 3 or 4, 5or 6, 2-4, 2 or 3, 2+3 setae, respectively, apex of processus terminalis with 3 setae, length of setae on segment III 0.003-0.005 mm, 0.11-0.22 times as long as basal diameter of the segment. Primary rhinaria ciliated, secondary rhinaria absent. Apex of rostrum reaching beyond middle coxae. Ultimate rostral segment wedge-shaped, apex dark brown ( Figures 15c , 16d ), 1.40-1.82 times as long as its basal width, 1.04-1.19 times as long as second hind tarsal segment, with 6 primary setae and 4 accessory setae.

*Thorax:* thoracic nota with C-shaped wrinkles. Venter with spinulous transverse rows. Legs short, distal parts of tibiae and tarsi brown,coxae with spinules ventrally, distal parts of femora with sparse and distinct imbrications, and distal parts of hind tibiae with weakly spinules, others smooth. Hind femora 1.13– 1.32 times as long as antennal segment III. Hind tibiae 0.31–0.38 times as long as body length, setae on hind tibiae short with acuminate to incrassate apices, 0.01–0.03 mm long, 0.50–0.83 times as long as middle diameter of the segment. First tarsal chaetotaxy: 3, 3, 3. Hind tibiae of nymph with spinules.

*Abdomen* : tergites I-VI with C- or O-shaped wrinkles, posterior areas of siphunculi, tergites VII and VIII with spinules. Venter with spinulous transverse rows. Spiracles nephroid, spiracular plates pale brown and slightly prominent. Setae on tergites I-VII similar to ones on head and thorax, tergite VIII with 4 long setae. Marginal setae on tergite Ι 0.0025 mm long, and spinal setae on tergite VIII 0.017–0.027mm long, 0.11–0.14 times and 0.88–1.38 times as long as basal diameter of antennal segment III, respectively. Siphunculi cylindrical, brown at apices, widest at base, slightly S-shaped, distinct imbricated and with a row of striate under developed flange ( Figures 15d , 16e ), 0.18–0.22 times as long as body, 2.79–3.75 times as long as its basal width, 2.25–2.40 times as long as cauda. Cauda pale brown, coniform, distal half slightly constricted ( Figures 15e , 16f ), 1.43–1.50 times as long as its basal width, with 5 setae. Anal plate pale brown, transverse oval, with 8–10 setae. Genital plate pale brown, broad round with 8–10 short posterior setae and 2 anterior setae.

***Alate viviparous female*** ( Figure 16g ) *.* Mounted specimens: Body pale brown. Body 1.33– 1.57 mm long, 0.60–0.61 mm wide. For general measurements see Table 3 .

*Head:* brown and smooth with sparse wrinkles dorsally and ventrally. Middle frontal tubercle indistinct, antennal tubercles low, slightly diverging at inner sides, with 2 pairs of antennal tubercles setae and a pair of median frontal setae. Dorsal setae of head short and acute, cephalic setae 0.007-0.017 mm long, 0.85-1.00 times as long as basal diameter of antennal segment III. Ventral setae similar to dorsal ones. Eyes large, with small ocular tubercles. Antennae 6-segmented, brown, inner margin of segments I and II sparse imbricated, segments III-VI imbricated ( Figures 15f , 15g , 15h ). Antennae 0.96-1.00 times as long as body, segment III slightly constricted, length in proportion of segments I-VI: 12-15, 11-14, 100, 48-51, 45-48, 33+68-76; processus terminalis 2.05-2.27 times as long as base of the segment. Primary rhinaria ciliated, segment III with 33-37 large non-protuberant circular secondary rhinaria, segment IV with 13 ones, segment V with 2-6 ones. Length of setae on segment III 0.007 mm, 0.38-0.43 times as long as basal diameter of the segment. Rostrum reaching middle coxae, ultimate rostral segment slender, wedge-shaped, apex dark brown, 2.55-3.25 times as long as its basal width, 1.89-2.00 times as long as second hind tarsal segments, with 6 primary setae and 4 accessory setae.

*Thorax:* legs long, brown except for basal parts of femora pale, coxae with spinules ventrally, distal parts of femora imbricated, and hind tibiae with very weakly spinules, others smooth. Hind femora 0.96-1.01 times as long as antennal segment III. Hind tibiae 0.56-0.63 times as long as body, setae on hind tibiae 0.012-0.020 mm long, 0.50-0.80 times as long as middle diameter of the segment. First tarsal chaetotaxy: 3, 3, 3.

*Abdomen:* tergites smooth. Abdominal tergites I and II with spinal and lateral sclerites fused into a broad brown stripe, tergites III-VI with a big brown patch, tergites II-VII each with a pair of marginal sclerites, marginal sclerites on tergites VI slightly bigger than others, marginal sclerites on tergites II-VII, and basal areas of each a pair of spinal setae on tergites III, IV and VI ornamented by spinules. Posterior areas of siphunculi, tergites VII and VIII with spinulose stripes. Dorsal setae slightly longer than ones of apterae. Marginal setae on tergite **I** 0.010 mm long, and tergite VIII with 4 long setae, spinal setae 0.03-0.04 mm long, 0.50-0.57 times and 1.25-2.43 times as long as basal diameter of antennal segment III, respectively. Siphunculi cylindrical, brown, widest at base, distinct imbricated and with a row of striate under developed flange ( Figure 15i ), 0.21-0.23 times as long as body, 4.00-4.96 times as long as its basal width, 3.44-3.68 times as long as cauda. Cauda pale brown, coniform, distal half slightly constricted, 1.20-1.48 times its basal width, with 5 setae. Anal plate transverse oval, brown, with 8 or 9 setae. Genital plate broad round and brown, with 12 short posterior setae and 2 anterior setae. Wing veins normal. Others are similar to apterae.

***Holotype** :* **China** . **Guizhou** : apterous viviparous female, Zunyi City (E107.1°, N28.2°), altitude 1470 m, 3.vi.2010, on *Plagiogyria japonica,* by X.M. Su (No. 24516).

***Paratypes** :* **China** . **Guizhou** : 1 alate viviparous female, Zunyi City, same data as holotype (No. 24510); 2 apterous viviparous females, 2 alate viviparous females, Zunyi City (E107.22°, N28.22°), altitude 854 m, 4.vi.2010, on *Plagiogyria japonica,* by X.M. Su (No. 24529); 3 apterous viviparous females, Zunyi City (E107.09°, N28.13°), altitude 1534 m, 5.vi.2010, on *Plagiogyria japonica,* by X.M. Su (No. 24547); **Tibet** : 7 apterous viviparous females, 2 apterous nymphs, Yadong County (E88.9°, N27.48°), altitude 2700 m, 23.vii.2005, on a kind of fern, by J.F. Wang (No. 16492).

***Host plant** : Plagiogyria japonica* (Plagiogyriaceae).

***Distribution** :* China (Guizhou, Tibet). ***Biology** :* The species infests *Plagiogyria japonica,* and usually colonizes loosely the distal part of the undersides of fronds ( Figure 17H ), and doesn’t cause any deformations to the hosts. The species have not been seen attended by ants.

***Comment:*** The new species is similar to the *Macromyzella polypodicola* ( Takahashi 1921 ), but differs from the latteras follows: (1) body dorsum with C-, or O-shaped wrinkles *(Macromyzella polypodicola:* body dorsum with reticulations), (2) antennae shorter than body, 0.51-0.63 times as long as body *(Macromyzella polypodicola:* antennae as long as or longer than body, 1.03-1.22 times as long as body), (3) siphunculi brown at apices and without reticulations under flange *(Macromyzella polypodicola:* siphunculi pale, with 4 or 5 rows of reticulations under the flange).
